# Strain-Engineered One-Dimensional van der Waals Heterostructures: BiTeI Confined in Carbon Nanotubes

**DOI:** 10.1021/acs.nanolett.6c02509

**Published:** 2026-08-25

**Authors:** Alejandro Cuesta-Troyano, Stefania Sandoval, Riccardo Rurali, Enric Canadell, Bernhard Dörling, Mariano Campoy-Quiles, Sofie Cambré, Sara Bals, Gerard Tobías-Rossell

**Affiliations:** † Institut de Ciència de Materials de Barcelona (ICMAB-CSIC), Carrer dels Til.lers, 08193, Cerdanyola del Vallès, Spain; ‡ Royal Academy of Sciences and Arts of Barcelona, Chemistry Section, La Rambla 115, 08002, Barcelona, Spain; § Theory and Spectroscopy of Molecules and Materials, Department of Physics, 198991 University of Antwerp, Universiteitsplein 1, 2610 Antwerp, Belgium; ∥ EMAT and Nanolight, Department of Physics, 198991 University of Antwerp, Groenenborgerlaan 171, 2020 Antwerp, Belgium

**Keywords:** 1D van der Waals, polar semiconductor, electron tomography, DFT, thermoelectricity, filling, encapsulation, sorting, ultracentrifugation, nanoreactor

## Abstract

Controlling the nanostructure and electronic properties of polar semiconductors could enable new applications in nanoelectronics. Here, we report the synthesis of one-dimensional BiTeI nanowires confined within single- and multiwalled carbon nanotubes using a sublimation-driven transport reaction. Aberration-corrected scanning transmission electron microscopy reveals highly ordered BiTeI nanowires ranging from one to six atomic layers. Density gradient ultracentrifugation enables the separation of filled and empty nanotubes, while Raman spectroscopy evidences host–guest interactions. Furthermore, the encapsulation of BiTeI leads to an increase in the optical band gap and a stable enhancement in electrical conductivity in macroscopic films. First-principles calculations show that compressive strain significantly modifies the BiTeI electronic structure, including reduced Rashba splitting and strain-induced band reordering. Overall, nanoconfinement is shown to be a powerful route to probe and engineer strain-tunable electronic states in polar semiconductors at the one-dimensional scale.

## Graphical abstract

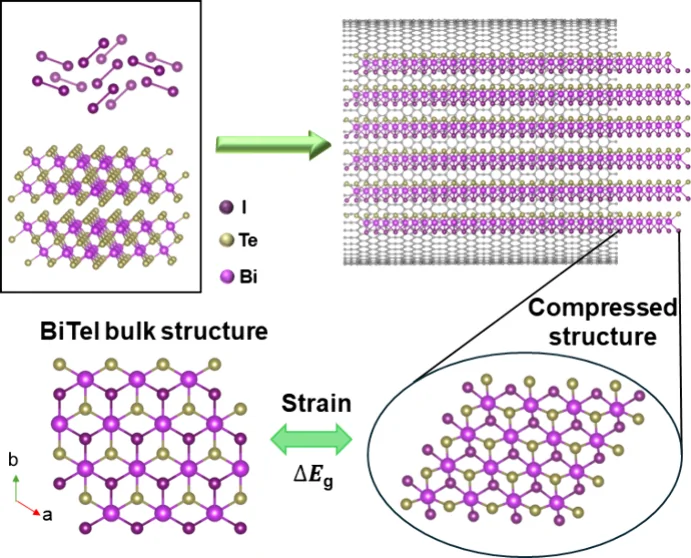

The interest in one-dimensional (1D) nanostructures has grown significantly in recent years due to the possibility of tuning the properties of bulk materials at the nanoscale.^1–3^ A subclass of these systems comprises 1D van der Waals heterostructures in which guest materials are confined within nanotubes, leading to modifications in structural, electronic, magnetic, and vibrational properties.^4–7^ The characteristics of these compounds can be tuned not only through their composition but also by controlling the structural parameters of the nanotubes that act as templates, such as chirality and diameter.^8,9^ Common hosts include transition-metal dichalcogenide (TMD) nanotubes, such as MoS_2_ and WS_2_,^10–12^ boron nitride nanotubes (BNNTs), and carbon nanotubes (CNTs). Among them, CNTs filled with metal halides have been widely investigated and find application as photodetectors, light emitters, and magnetic memories and in biomedical imaging.^13,14^ On the other hand, the direct band gap exhibited by TMD monolayers makes them highly attractive for optoelectronic applications such as detectors, LEDs, and transistors.^15^ Not surprisingly, also the combination of TMDs with CNTs has been studied.^16,17^ In these structures, the tunable electronic and spin–orbit properties of layered chalcogenides make their interfacial coupling particularly appealing.^18,19^

Bismuth telluroiodide (BiTeI) is a polar noncentrosymmetric semiconductor with strong spin–orbit coupling, being a promising candidate for topological insulator applications owing to its large Rashba effect.^20,21^ In addition, BiTeI exhibits thermoelectric performance with reported figure of merit (ZT) values of 0.10 at 300 K and 0.4 at 570 K.^22^ Unlike Bi_2_Te_3_, whose centrosymmetric structure suppresses spin-splitting effects and exhibits a weaker Rashba effect, BiTeI displays a much stronger spin–orbit coupling. Structurally, Bi_2_Te_3_ consists of quintuple layers (Te–Bi–Te–Bi–Te), whereas BiTeI is composed of triple layers (I–Bi–Te).

Several theoretical studies have predicted remarkable piezoelectric properties in monolayer BiTeI,^23,24^ as well as notable changes in Rashba-type band splitting when reducing the dimensionality from bulk to few-layer systems.^25^ Experimentally, Fülöp et al.^26^ demonstrated the exfoliation of BiTeI down to 50–100 nm, while Petrini et al.^27^ reported the formation of monolayer domains. Despite these advances, the behavior of BiTeI under 1D confinement remains unexplored and has been the focus of the present study.

BiTeI nanowires were synthesized by reacting Bi_2_Te_3_ and I_2_, using CNTs as templates (Figure S1). The formation of BiTeI crystals was only observed in the presence of CNTs, demonstrating that nanoscale confinement enables unique reaction pathways (see SI for details, Figure S2). The successful encapsulation and crystallization of BiTeI within MWCNTs were initially confirmed by HAADF-STEM analysis. The intensity using this technique scales with the atomic number (Z) of the elements present in the sample as well as with the sample thickness.^28^ Low-magnification HAADF-STEM images revealed a high degree of filling within the MWCNTs, as shown in Figure S3a–c. Each bright line observed in the images corresponds to BiTeI encapsulated inside MWCNTs, as confirmed by EDX analysis. Statistical analysis revealed a BiTeI filling yield of 66.8%, which is notably high compared with typical values for filled MWCNTs. The number of layers is governed by the internal diameter of the host MWCNT, which acts as a size-limiting template (Figure S4).

Figure S5 shows the HAADF-STEM image along with an EDX elemental map of a nanowire composed of five BiTeI layers confined within an MWCNT. As expected, bismuth, tellurium, and iodide are all colocated within the same spatial region of the nanowire. The EDX spectrum (Figure S6) presents a clearly differentiated signal at 10.8 keV corresponding to Bi-Lα. Tellurium and iodide peaks appear at similar energies but can nevertheless be discerned (Te-Lα at 3.78 keV and I-Lα at 3.94 keV).

XPS analysis of the BiTeI@MWCNT sample (Figure S7) was performed to provide further evidence of the sample composition and to assess the interactions between the different elements. In the I 3d region, the characteristic iodide peak (619.1 eV) is accompanied by two satellite peaks (617.6 and 620.4 eV), likely arising from the interaction of iodide with the CNTs.^14^ The Te 3d region reveals a peak at 572.6 eV corresponding to tellurium anionic species bonded to bismuth. The 573.6 eV peak has been previously assigned to Te^0^ and Te^2–^, while the 576.4 eV signal corresponds to a Te^4+^.^29–31^ The presence of different coordination environments might arise from slight stoichiometric deviations at the edges of the nanowires or charge transfer with the CNT. The Bi 4f region shows peaks at 157.8 and 159.2 eV, corresponding to Bi^3+^ bonded to tellurium and iodide, respectively.^32^ A minor peak at 158.2 eV may indicate the presence of an altered bismuth coordination or the interaction with the CNT.^33^ Thus, XPS data confirm the elemental composition of the sample and reveal clear host–guest interactions.

The HAADF-STEM image in Figure 1 shows a BiTeI nanowire confined within an MWCNT, consisting of six atomic layers and exhibiting a diameter of about 5 nm. The image reveals atomic columns with varying contrast, where the highest intensity columns contain bismuth atoms (Z = 83), and tellurium (Z = 52) and iodide (Z = 53) atoms appear slightly dimmer. The brightest atomic columns are located at the center of the layers, consistent with the bulk crystal structure of BiTeI.^22^ The intensity profile along direction 1 indicates an in-plane Bi–Bi distance of 0.22 ± 0.01 nm, which is in excellent agreement with the structure of the bulk phase.^22^ On the other hand, the average interlayer spacing determined from the intensity profile along direction 2 turned out to be 0.73 ± 0.04 nm (2.19 nm for three layers), close to the interlayer spacing of the bulk, 0.72 nm.^22^ The difference between the outer and central atomic positions within each trilayer can also be resolved in the high-resolution image. The Bi–Te and Bi–I projected distances along the [0 1 0] direction are measured at 0.17 ± 0.01 nm and 0.21 ± 0.01 nm, respectively. These are also in excellent agreement with the projected distances depicted in Figure 1e for the bulk structure (0.17 and 0.21 nm), being the bond lengths of Bi–Te (0.304 nm) and Bi–I (0.372 nm).^22,26^

**Figure 1:**
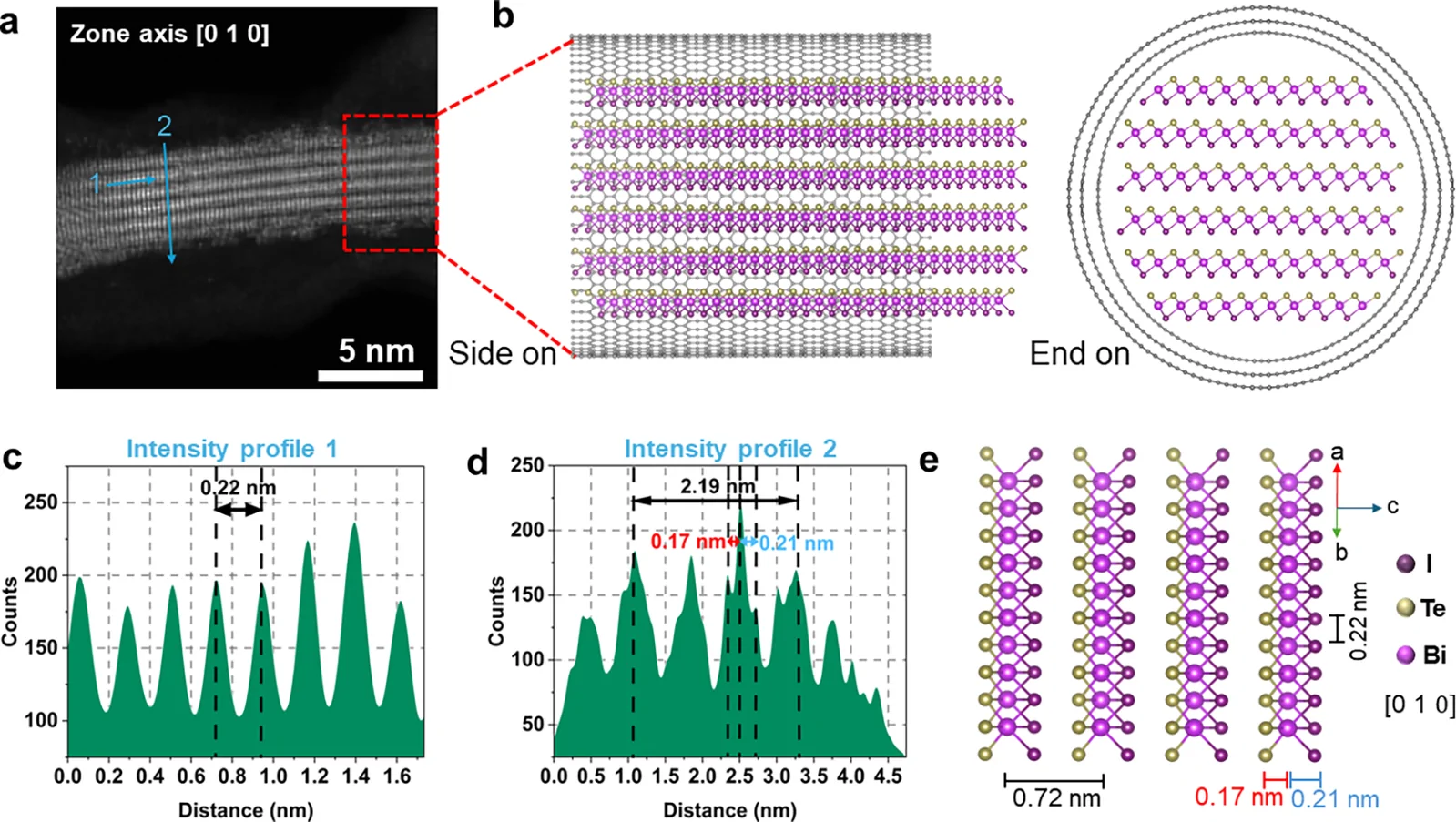
Structural characterization of a BiTeI nanowire encapsulated within an MWCNT. (a) HAADF-STEM image of BiTeI@MWCNT along the [0 1 0] zone axis. (b) Schematic representation of the BiTeI nanowire confined within the nanotube cavity (side-on and end-on views); I, Te, and Bi atoms are represented by purple, ochre, and violet spheres, respectively. (c, d) Intensity profiles along blue arrows 1 and 2 (in image a). (e) Bulk crystal structure of BiTeI; projected distances are indicated for ease of comparison with the intensity profiles.^22^

The structural characterization of another BiTeI nanowire is presented in Figure S8. In this case, the nanowire is imaged along the [1 0 3] zone axis. The hexagonal pattern observed on this projection enables a direct comparison between the measured spacings (intensity profiles 1 and 2) and the bulk BiTeI structure, depicted in the structural model (Figure S8b). The measured lattice parameters in the HAADF-STEM image reveal slight deviations from the crystal structure of bulk BiTeI,^22^ with measured spacings of 0.44 and 0.40 nm, along the *b* and *a* axes, respectively, compared to the reference values of 0.45 and 0.41 nm.

Electron tomography was next employed to determine the different morphologies of the 1D vdW heterostructures. As demonstrated in Figure 2, the encapsulated BiTeI reveals a high degree of crystalline order. The achieved resolution allowed for the direct visualization of individual Te–Bi–I atomic columns, confirming the preservation of the trigonal ‘sandwich’ motifs under 1D confinement. The characteristic lateral displacement between layers is clearly observable in the reconstructed volume. Furthermore, top-view analysis indicates a slight bending of the layers, a structural deformation likely induced by the radial pressure of the CNT template, although minor contributions from reconstruction artifacts due to the limited tilt range cannot be entirely ruled out. These findings are further supported by the analysis of the heterostructure shown in Figure S9, which likewise exhibits a five-layer BiTeI nanowire. In this instance, oblique slices along the xz plane revealed a localized dislocation in one of the layers, highlighting the presence of structural defects. The outer layers of this nanowire also show a curvature-driven bending effect. Full 3D dynamics and slice-by-slice morphology of these structures are provided in Supporting Videos S1 and S2.

**Figure 2:**
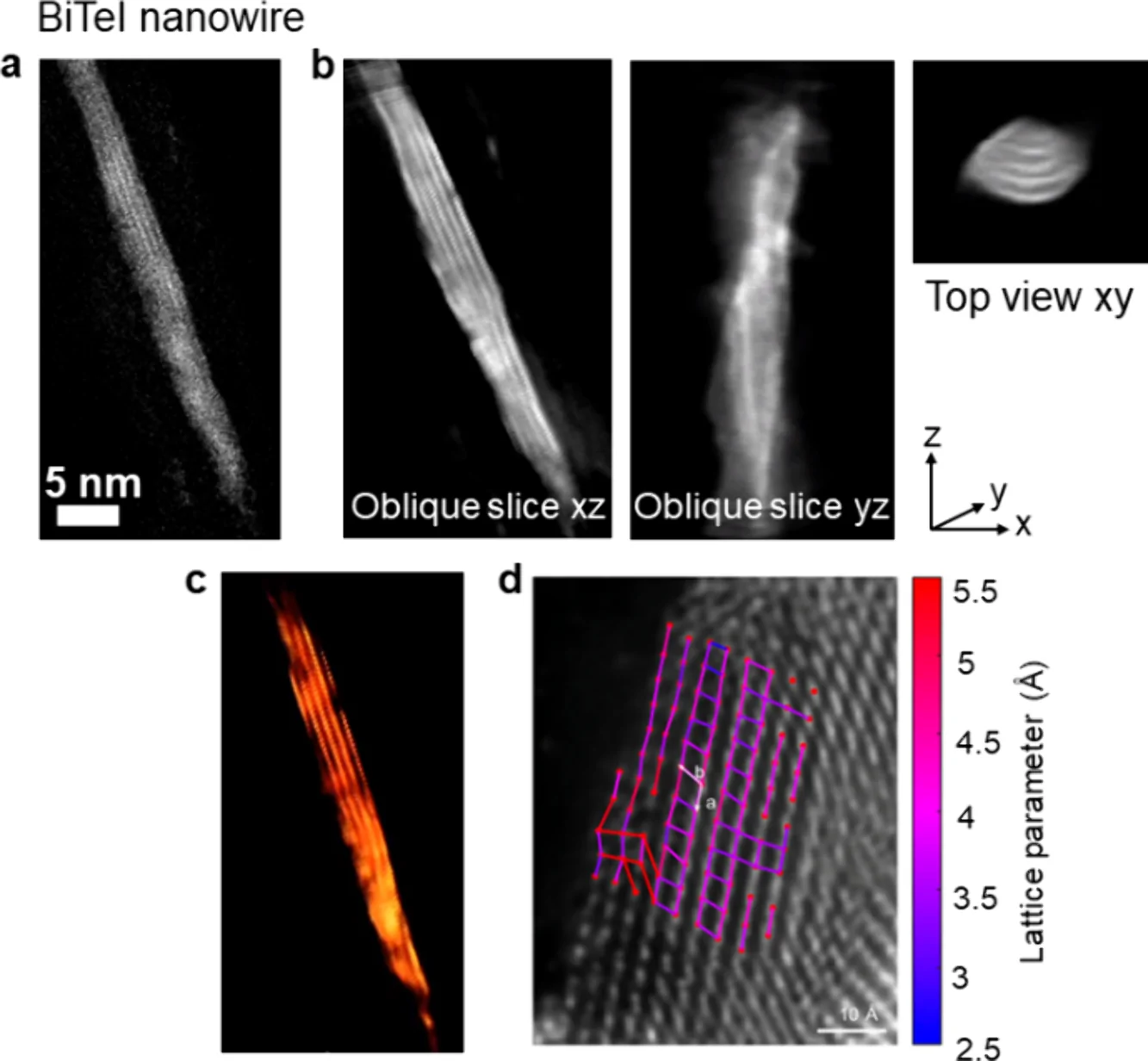
Electron tomography reconstruction of a BiTeI nanowire encapsulated within a MWCNT. (a) HAADF-STEM projected image obtained prior to tomographic reconstruction. (b) Oblique slices of the tomographic volumes along the yz, xz, and xy planes, revealing the spatial distribution and morphology of BiTeI within the MWCNT cavity. (c) Corresponding 3D reconstruction showing the internal structure and morphology of the encapsulated material. (d) HAADF-STEM image with overlapping strain analysis of a BiTeI nanowire encapsulated within an MWCNT. The lattice strain along the a and b directions was quantified through atomic position refinement using the StatSTEM software.

Overall, electron tomography provides valuable insights into the 3D atomic arrangement of the BiTeI nanowires within the CNTs and allows a deeper understanding of structural changes induced by confinement.

Lattice parameter analysis of an additional BiTeI-filled MWCNT further demonstrates significant unit cell deformations, confirming that the structural confinement alters the BiTeI lattice (Figure 2d). This confinement generates a biaxial asymmetric compressive strain, calculated as ε_*x*_ = −0.06 and ε_*y*_ = −0.198. Such deviations from the equilibrium lattice constants suggest a profound modification of the material’s internal energy. Consequently, these structural perturbations are expected to alter the electronic and vibrational properties of the encapsulated 1D nanostructure compared to its bulk counterpart, potentially leading to emergent phenomena not observed in the unconfined phase.

The same protocol used for the synthesis of BiTeI@MWCNTs was used to encapsulate BiTeI within SWCNTs. As shown in Figure 3, BiTeI is also observed to crystallize within the SWCNTs. The strong spatial confinement imposed by the nanotube diameter restricts the encapsulated BiTeI to one or two layers. A high proportion of filled SWCNTs can be readily observed by HAADF-STEM, but the presence of bundles limits the accurate quantification of the filling ratio. EDX elemental mapping and spectrum (Figure S6c,d) confirmed the presence of Bi, Te, and I. EELS measurements corroborated the presence of Te and I, showing Te–M_4,5_ and I–M_4,5_ edges at 620.2 eV and 649.9 eV, respectively, which is coherent with reported values for BiTeI.^34^ The Bi edge was not detected, probably due to the limited signal-to-noise ratio at high energy losses, where Bi-related features are expected. This limitation is commonly encountered in EELS analyses of confined nanostructures, where the scattering volume is small and high-energy edges are inherently weak.

**Figure 3:**
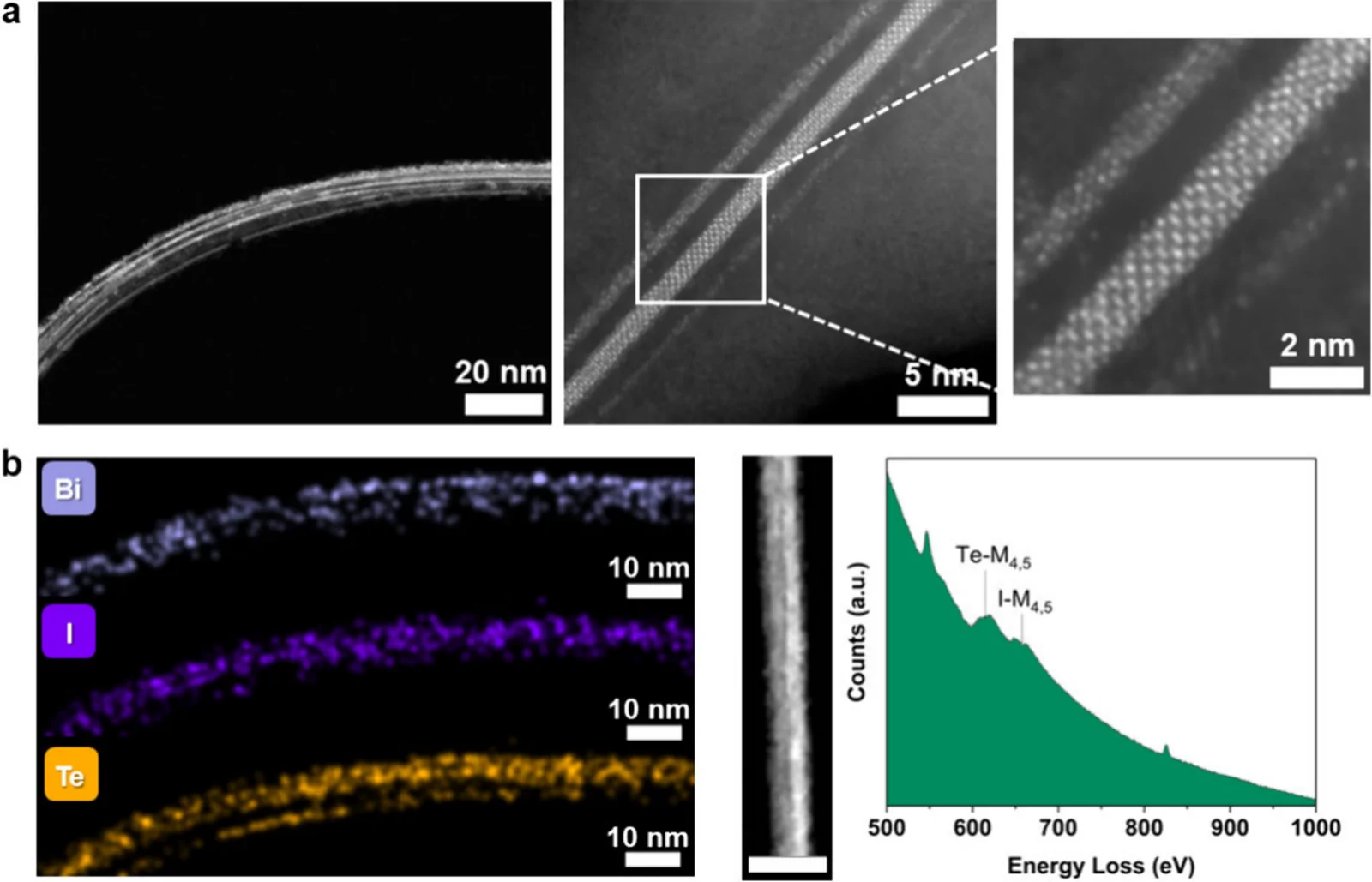
Structural and chemical characterization of BiTeI confined within SWCNTs. (a) HAADF-STEM images at increasing magnification showing SWCNTs filled with BiTeI. (b) EDX elemental maps confirming the presence and colocalization of Bi (light violet), I (purple), and Te (orange) and EELS spectrum acquired in the region shown in the HAADF-STEM image, displaying Te-M_4,5_ and I-M_4,5_ edges. The scale bar in the HAADF-STEM image corresponds to 10 nm.

The analysis performed so far demonstrates the affinity of BiTeI for the carbon hosts, likely due to the minimal electronegativity difference between bismuth and its counterions, tellurium, and iodide.^12^ This interaction appears particularly pronounced for SWCNTs, whose straighter morphology seems to promote a more efficient nanowire growth,^35^ based on visual inspection of HAADF-STEM images (Figure S3d–f). CVD-grown MWCNTs tend to have kinks, bends, and other structural defects that might interfere in the crystallization of materials. The use of templates with varying internal diameters enables the formation of nanowires with different layer numbers, thereby providing control over the dimensions of the inorganic material. Consequently, the properties of confined compounds can be systematically tuned.

Density gradient ultracentrifugation (DGU) was employed to separate individual nanotubes and distinguish between empty and BiTeI-filled SWCNTs (Figure S10). Prior to DGU, the filled sample exhibited a Raman G-band shift of 0.45 cm^–1^ compared to empty reference SWCNTs (Figure S11). The upshift of the G-band can arise from a weak charge transfer^36,37^ or can be an effect of strain.^38^ Our current results cannot disentangle both effects, but do indicate they are weak and much smaller than the ones observed for other systems.^36–38^ Therefore, we conclude that there is only a very weak interaction between the encapsulated species and the SWCNT host. In this work, Raman spectroscopy proved to be particularly effective for monitoring both the separation process and the host–guest interaction, especially in the radial breathing mode (RBM) region.

Figure 4 shows Raman spectra measured in low-dispersion mode for BiTeI@SWCNTs and empty reference nanotubes from fractions B and C after DGU. In the G^+^-band, fraction B exhibits a signal at 1589.5 cm^–1^, identical for both the reference and the BiTeI-filled samples. This suggests a lack of significant charge transfer or internal strain, which, combined with the fraction’s characteristic density observed during DGU, indicates that this fraction is predominantly composed of either unfilled SWCNTs or nanotubes filled with the aqueous medium. In contrast, fraction C of the filled sample shows a shift to 1590 cm^–1^, demonstrating that it is enriched in BiTeI@SWCNTs. These observations confirm the effectiveness of the DGU separation, as evidenced by the distinct Raman shift observed in fraction C of the BiTeI@SWCNT sample relative to the other fractions. These shifts are attributed to charge transfer^3,39,40^ or mechanical stress^41,42^ induced by the encapsulation, in agreement with previous observations for Bi_2_Te_3_- and Bi_2_Se_3_-filled SWCNTs.^43^

**Figure 4:**
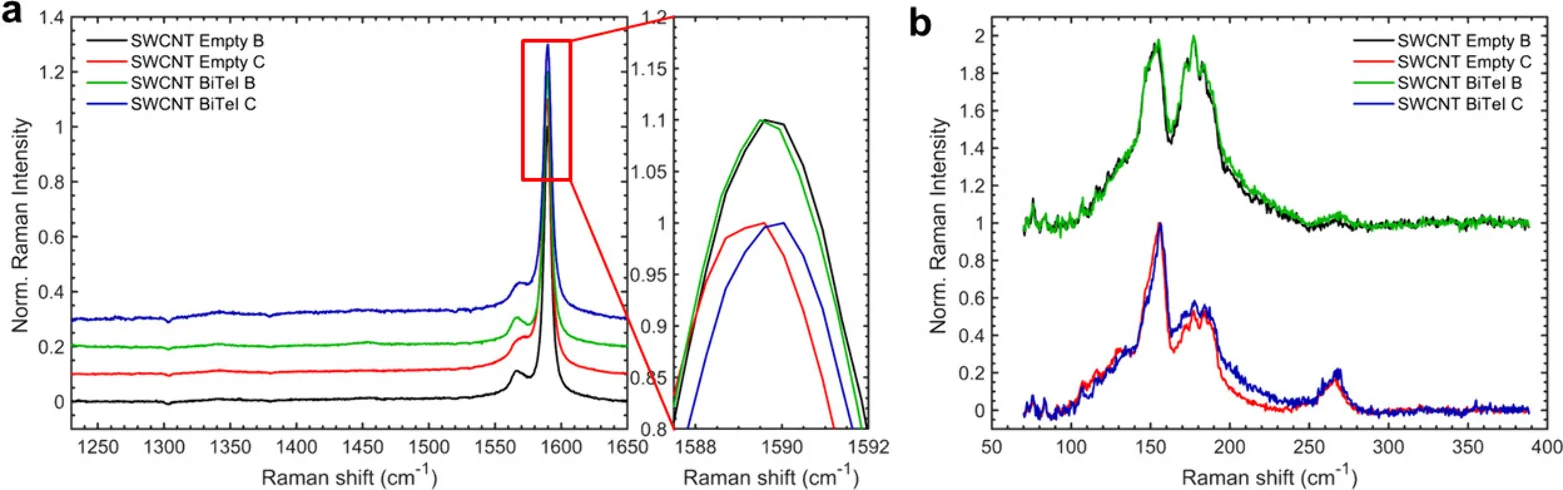
Raman spectra of empty and BiTeI-filled SWCNTs excited at 514.5 nm after the DGU process. (a) G band region showing the G^+^ and G^–^ components, with a magnified view of the G^+^ peak revealing slight shifts upon filling. (b) Radial breathing mode (RBM) region, highlighting differences between empty and filled nanotubes in the low and high Raman shift ranges, consistent with the encapsulation of BiTeI inside SWCNTs. Spectra are normalized and vertically offset for clarity.

The RBM region allows for the identification of specific nanotube populations based on their diameter as the Raman shift is inversely proportional to the tube size.^44,45^ The spectra reveal a prominent, broad band between 145 and 160 cm^–1^, which originates from the primary SWCNT population with an average diameter of approximately 1.5 nm.

Consistent with the trends observed in the G band, the RBM signatures differentiate the filling efficiency across the various samples. Fraction B of the BiTeI@SWCNT sample and fractions B and C of the empty sample exhibit an identical Raman shift at 155.1 cm^–1^. In contrast, fraction C of the BiTeI@SWCNT sample displays a noticeable shift to 156.2 cm^–1^. This subtle blueshift provides further evidence of the mechanical and electronic host–guest interaction, confirming that successful encapsulation is uniquely localized within fraction C.

To isolate the vibrational signature of the encapsulated BiTeI, the empty fraction C signal was subtracted from the encapsulated fraction C. The resulting difference spectrum, presented in Figure S12, reveals characteristic peaks at 95 cm^–1^ and 120 cm^–1^, which are assigned to the A_1_ and E vibrational modes of BiTeI.^46,47^ Furthermore, emerging spectral features are detected at 200 cm^–1^ and 290 cm^–1^. These high-frequency shifts are not present in the bulk material and are tentatively attributed to the strong 1D spatial confinement and potential lattice distortions imposed by SWCNTs. This analysis confirms the effectiveness of DGU, as BiTeI-filled nanotubes exhibit a higher density that enables their partial separation from nonfilled (or water-filled) nanotubes. Raman spectroscopy of the G-band region further reveals the interaction and doping effect between BiTeI and SWCNTs.

To assess the impact on the macroscopic properties of the hybrid, the thermoelectric response was investigated. Compared to a simple mixture, encapsulation can serve as an avenue to change the doping in a potentially more stable manner.^48–50^ Additionally, BiTeI shows a respectable thermoelectric performance, characterized by a Seebeck coefficient (*S*) of −81 μ·V·K^–1^, an electrical conductivity (σ) of approximately 323 S·cm^–1^, and a low lattice thermal conductivity (k_L_) of 1.009 W·m^–1^·K^–1^.^51^ With that in mind, we characterized both σ and *S* of BiTeI@SWCNT films (Figure 5). MWCNTs exhibit over an order of magnitude lower σ than and similar *S* to SWCNTs and were not further investigated for thermoelectric applications. Samples were stored in air, and measurements were repeated over time, to estimate stability. Compared to the reference films of unfilled, but otherwise identically treated SWCNTs, films of BiTeI@SWCNT do not show a significantly different *S*. On the other hand, σ increased noticeably, if to a limited degree, compared to more traditional dopants.^48,50^ While the increase in σ upon filling may not be particularly large, it can be considered stable, since over the investigated time frame of about 250 days, no significant changes were observed. The observed change in σ could be a combination of several factors. Possible contributions include charge transfer, although it would be rather limited according to Raman spectroscopy and the lack of a significant change in the Seebeck coefficient. This limited charge transfer may be related to the difficulty to obtain a type II heterojunction between the CNT and BiTeI given the small bandgap of the latter (as determined below). Conductivity changes may also be due to modifications to the CNT electronic structure induced by BiTeI or due to alterations to the CNT transport network, even though we tried to minimize their effect by subjecting both filled and unfilled samples to exactly the same processing steps.

**Figure 5:**
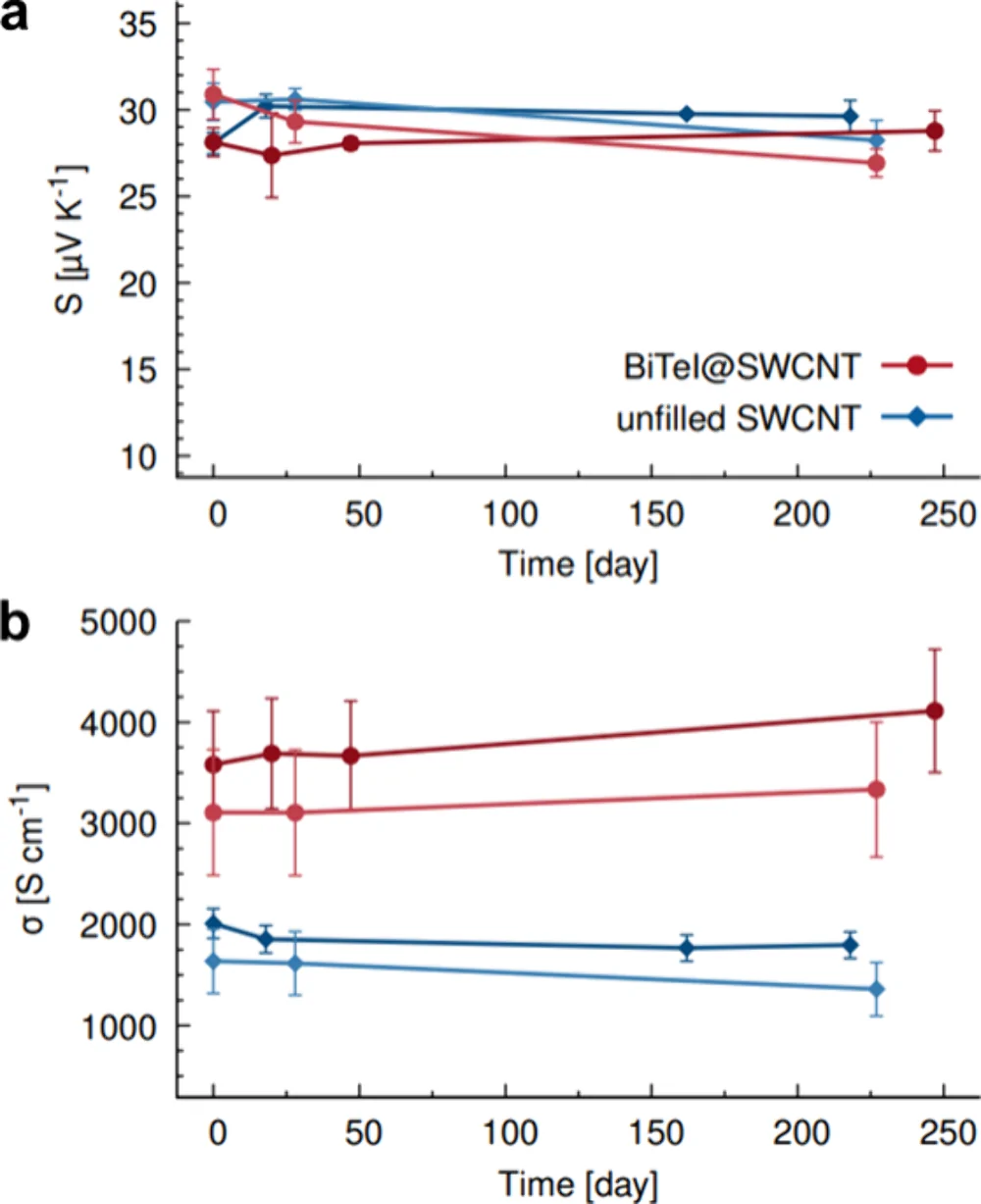
Evolution of (a) the Seebeck coefficient and (b) the electrical conductivity of two BiTeI-filled SWCNT films (red circles) and two unfilled reference samples (blue diamonds) over time.

To assess how the observed BiTeI structural strain influences the electronic structure of the encapsulated BiTeI, optical E_g_ were estimated from the absorption spectra using Tauc plots.^51,52^ Figure S13 illustrates the photon energy (*hν*) dependence of (A*·* *hν*)^2^ for both pristine and filled CNT samples, as well as the subtracted signal corresponding to the encapsulated BiTeI. A direct transition was assumed for the analysis, as the wave vector difference near the band gap is negligible for the Rashba spin-split BiTeI system. The optical band gap was determined by the linear extrapolation of the high-energy region to the abscissa, following the relation

$${\left(A\cdot \mathrm{h\nu}\right)}^{2}=A\left(\mathrm{h\nu}‐E_{g},\mathrm{optical}\right)$$

The results indicate values that differ from the bulk band gap of BiTeI (∼0.33 eV).^53^ For BiTeI@SWCNT, the measured E_g_ was 0.86 ± 0.01 eV, while the isolated contribution of the confined BiTeI yielded 0.94 ± 0.05 eV. In the case of BiTeI@MWCNT, the values were slightly lower, at 0.75 ± 0.01 eV for the composite and 0.80 ± 0.03 eV for the encapsulated material alone. This increase in the band gap is consistent with theoretical models which predict that spatial constraints imposed by the host material’s reduced dimensionality lead to a significant increase in E_g_. Notably, the higher E_g_ values recorded for SWCNT-encapsulated BiTeI compared to the MWCNT counterparts correlate with a reduced number of BiTeI layers, dictated by the narrower internal diameter of the single-walled templates. The confinement-induced increase of the optical band gap to ∼0.8 eV places this hybrid material in an energy range of particular interest for near-infrared optoelectronics. Such a band gap is well suited for infrared photodetection and optical sensing at technologically relevant wavelengths and could also be explored for infrared imaging and thermal sensing applications. Moreover, such a gap would also be compatible with the third telecommunication spectral window, devoted to large distance communication using fiber optics, such as submarine intercontinental lines.

Because of the large strain observed in our samples and the induced change in the optical gap, we deemed it worthwhile to perform a model study of the basic variations in the electronic structure and Rashba effect that may be induced under strain. To make it as general as possible, we will consider in detail a single BiTeI layer. The effect of charge transfer and the increase in the number of layers can be subsequently introduced by varying the Fermi level position and decreasing the separation between the valence and conduction bands, respectively. We thus have carried out density functional theory (DFT) calculations with van der Waals corrections and full geometry optimization (see computational details) on a single BiTeI layer under different uniform and nonuniform biaxial compressive strains. The optimized structural parameters for the single layer are *a* = 4.383 Å, Bi–Te = 3.068 Å, and Bi–I = 3.286 Å, in good agreement with the experimental bulk structure: *a* = 4.339 Å, Bi–Te= 3.039 Å, and Bi–I = 3.273 Å.^22^ Compared with the optimized bulk structure, the *a* parameter of the single layer increases by 0.48% and the Bi–Te/Bi–I bond lengths change by +0.06%/–0.81%. The calculated band structures for the single layer and the bulk, which are in good agreement with previous calculations,^20,26,54–56^ are reported in Figures S14 and S15.

The Rashba effect in bulk BiTeI is due to the band splitting induced around the A point (0, 0, c*/2) of the Brillouin zone or the Γ point (0, 0, 0,) for the single layer. Shown in Figure 6a is the evolution of the top of the valence and bottom of the conduction bands along the Γ–M direction for different values of the uniform biaxial compressive strain (ε = 0.00 to −0.14). For the unstrained single layer, we find a Rashba energy (E_R_, i.e., the difference between the bottom of the conduction band and the energy at Γ) of 39 meV and the minimum of the conduction band occurs at *k*_0_ = 0.044 Å^–1^. From these values, a Rashba parameter of α_R_ = 1.76 eV·Å is obtained. The energy gap (E_g_) between the valence and conduction band is found to be 0.70 eV. The values for bulk BiTeI are α_R_ = 4.43 eV·Å and E_g_ = 0.22 eV, making clear the strong control of the interlayer interactions over the band gap and Rashba effect.

**Figure 6:**
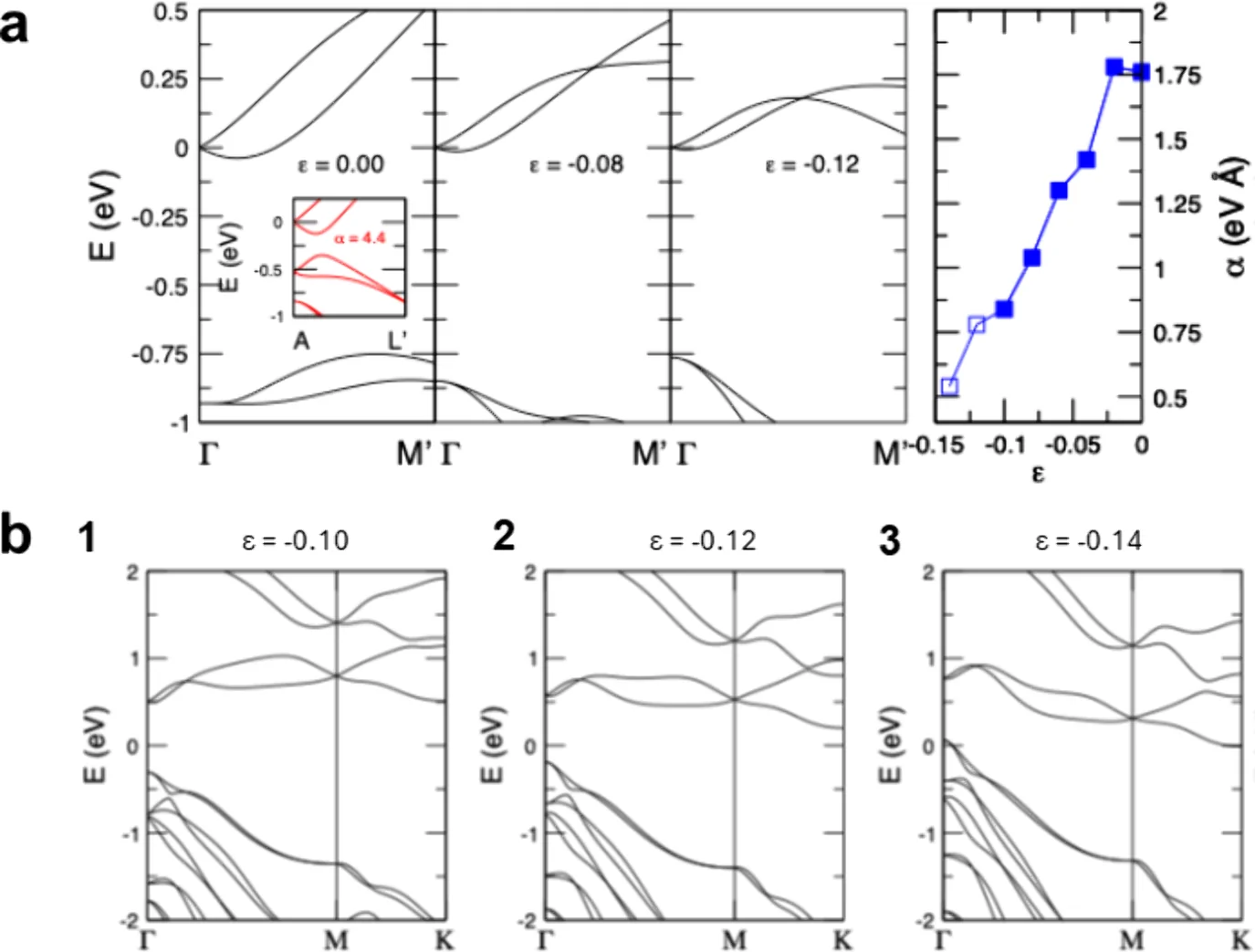
(a) Electronic band structure of single-layer BiTeI for three different degrees of biaxial compressive strain (left: ε = 0.000, the unstrained sample; middle: ε = −0.08; right: ε = −0.12) along the Γ–M high-symmetry direction (M′ is a *k*-point at 1/3 of the segment connecting Γ to M). The inset displays the full band structure along the A–L direction (L′ is a *k*-point at 1/3 of the segment connecting A to L). See the Supporting Material for the band structure of the bulk, single layer and some single layers with nonuniform strain—where compression along the *y*-axis is larger than the one along the *x*-axis—in the full Brillouin zone. On the right-hand side we display the Rashba factor α_R_ = 2ΔE/*k*_0_ (see text) as a function of strain: the data points for the largest compressive strains, ε = −0.12 and −0.14, are indicated for the sake of completeness, but they are not significant, because at those strain levels the valence band minimum is no longer around Γ (see below for the corresponding discussion in the text). (b) Electronic band structure of single-layer BiTeI for three different degrees of biaxial compressive strain, (1) ε = −0.10, (2) ε = −0.12, and (3) ε = −0.14, where Γ= (0, 0), M = (a*/2, 0), and K = (a*/3, a*/3).

As the uniform biaxial strain is introduced, two trends are clearly visible in Figure 6a. First, both E_R_ and *k*_0_ decrease, although the decrease of the Rashba energy dominates, so that for ε ∼ −0.10 the Rashba parameter has been reduced to around one-half (right panel). Second, when strain increases, the separation between the two split conduction bands decreases and around ε ∼ −0.06 the two bands cross. After this crossing, the two bands continue going down in energy, and finally, for a strain ε ∼ −0.12, they are lower in energy at the M point than at the Γ point (Figure 6b2). Remarkably, when moving from the M point toward the K point, the lower of the two bands still goes down in energy and in fact overlaps in energy with the top of the valence band at Γ (Figure 6b3). In that case, the system is entering a genuinely semimetallic or metallic regime. In fact, already for ε ∼ −0.12 the bottom of the valence band does not occur around Γ but between M and K (Figure 6b1). In this region, the splitting between the two bands varies considerably, and thus the Rashba parameter can also vary quite strongly. Consequently, this model study suggests that inducing uniform biaxial strain in the single layer may lead to an important variation in the physical behavior. Note that the metallic state induced by the compressive strain is a peculiar one. Since only one of the two split conduction bands becomes filled, the electron carriers are spin-polarized. Although the total spin will be nil because of the compensation at the ±*k*_0_ regions, the Fermi surface will exhibit a spin-polarization. We also considered the case of a nonuniform biaxial compressive strain. Shown in Figure S16 are the results for the case where ε_*y*_ = 2ε_*x*_ (i.e., for a ratio not far from our results within the nanotubes). In general, the results follow the same trend although more slowly. For instance, the case for ε_*x*_ = −0.04/ε_*y*_ = −0.08 is quite similar to that for ε = −0.04 for uniform biaxial strain. In addition, the real crossing between the two bands for the uniform biaxial strain becomes an avoided crossing for the nonuniform case, because of the disappearance of the symmetry planes perpendicular to the layers containing the a* axis. In short, nonuniform biaxial compressive strain induces a similar but softer evolution of the single-layer electronic structure.

In summary, the strain generated in single layers of BiTeI can provide a significant variation in its conduction band. The three pairs of empty bands are mainly built from the three Bi 6p levels, which mix in an antibonding way with the Te 5p levels. At Γ, the conduction band is mostly based on the Bi 6p_*z*_ levels (i.e., the p levels perpendicular to the layer plane), but when going away toward the M and K points, there is an avoided crossing with an upper band based on the Bi 6p_x,y_ levels (i.e., the p levels within the plane of the layer) and it is strongly stabilized from Γ to M/K. Such interchange in the conduction band orbital character is responsible for the possible anomalous behavior of strained BiTeI layers for substantial compressive strain. We thus suggest that the physical behavior of BiTeI single or multilayers can be substantially modified by varying the size of the nanotubes. Of course, when interlayer interactions are introduced in bilayers, trilayers, etc., the separation between the valence and conduction band decreases, and this anomalous conduction band can come into play with weaker compressive strains and can probably be easier to observe.

We have demonstrated that CNTs act as nanovessels for the synthesis of BiTeI, resulting in 1D van der Waals heterostructures with high filling efficiency. The encapsulated material experiences significant compressive strain imposed by the confinement. DFT calculations indicate that strain tunes the electronic structure, including reduced Rashba splitting and band gap, and may drive the system toward a semimetallic or metallic regime. Spectroscopic measurements evidence host–guest interactions and a significant increase in the optical band gap, while density gradient ultracentrifugation enables the isolation of filled nanotubes. At the macroscopic level, BiTeI-filled SWCNT films exhibit a stable increase in electrical conductivity, demonstrating a controlled-doping strategy. Nanoconfinement provides a powerful route to accessing and engineering strain-tunable electronic properties in low-dimensional polar semiconductors.

## Supplementary Material







## Data Availability

The data underlying this study are openly available in DIGITAL.CSIC at 10.20350/digitalCSIC/20225.

## Notes

Experimental section, characterization, computational methods, schematic diagram of the PVT system (Supporting Figure 1), morphological and chemical characterization of Bi_2_Te_3_ bulk material (Supporting Figure 2), HAADF-STEM images of BiTeI@MWCNTs and BiTeI@SWCNTs (Supporting Figure 3), high-resolution HAADF-STEM images of different layered BiTeI nanowires (Supporting Figure 4), EDX elemental mapping of BiTeI@MWCNTs (Supporting Figure 5), EDX spectrum of BiTeI@MWCNTs and BiTeI@SWCNTs samples (Supporting Figure 6), XPS spectra of BiTeI@MWCNT (Supporting Figure 7), structural characterization of a BiTeI@MWCNT (Supporting Figure 8), electron tomography reconstruction of BiTeI@MWCNT (Supporting Figure 9), schematic diagram of the DGU process (Supporting Figure 10), Raman spectra of empty SWCNTs and BiTeI@SWCNTs (Supporting Figure 11), Raman spectrum RBM subtracted from BiTeI@SWCNT (Supporting Figure 12), Tauc plots for MWCNT and SWCNT unfilled and filled samples (Supporting Figure 13), full electronic band structure of single-layer BiTeI (Supporting Figures 14 and 15), evolution of electronic bands as a function of nonuniform strain (Supporting Figure 16) (PDF)
3D reconstruction by electron tomography of a BiTeI nanowire encapsulated within an MWCNT (MPG)
High-resolution 3D reconstruction by electron tomography of a BiTeI nanowire encapsulated within an MWCNT (MPG)
