# Supplementary material for: Strain-Engineered One-Dimensional van der Waals Heterostructures: BiTeI Confined in Carbon Nanotubes
Source: Nano Lett. 2026 Aug 25;26(35):11738–47. doi: 10.1021/acs.nanolett.6c02509 (PMC13564961; doi:10.1021/acs.nanolett.6c02509)
Supplement: Supplementary file 1 [file nl6c02509_si_001.pdf]

Supporting Information

Strain-Engineered One-Dimensional van der Waals Heterostructures: BiTeI Confined in Carbon Nanotubes

Alejandro Cuesta-Troyano[a], Stefania Sandoval[a], Riccardo Rurali[a], Enric Canadell[a,b], Bernhard Dörling[a], Mariano Campoy-Quiles[a], Sofie Cambré[c], Sara Bals[d], Gerard Tobías-Rossell[a*]*

[a]Institut de Ciencia de Materials de Barcelona (ICMAB-CSIC), Carrer dels Til.lers, s/n 08193 Cerdanyola del Vallès, Spain

[b]Royal Academy of Sciences and Arts of Barcelona, Chemistry Section, La Rambla 115, 08002 Barcelona, Spain

[c]Theory and Spectroscopy of Molecules and Materials, Department of Physics, University of Antwerp, Universiteitsplein 1, 2610 Antwerp, Belgium

[d]EMAT and Nanolight, Department of Physics, University of Antwerp, Groenenborgerlaan 171, 2020 Antwerp, Belgium

*Corresponding authors: sofie.cambre@uantwerpen.be; gerard.tobias@icmab.es

Experimental section

Synthesis of BiTeI within carbon nanotubes

MWCNTs grown by chemical vapor deposition (CVD) were steam-treated in order to open the ends, remove amorphous carbon and residual graphitic particles.[1] The sample was subsequently refluxed in 6 M HCl for 5 h to dissolve catalytic metal nanoparticles remaining from the MWCNTs synthesis. After acid treatment, the material was collected by filtration, as a black solid powder on top of a 0.2 µm hydrophilic PTFE membrane (Omnipore™, 47 mm diameter, Merck Millipore), and dried overnight. SWCNTs (eDIPS EC1.5P, CVD, 1.5 – 2.5 nm diameter, Meijo Nano Carbon) were used as received, having been already purified by the supplier.

Carbon nanotubes were filled with BiTeI using Bi_2Te_3 and I_2 as precursors. Sample handling was performed inside an argon-filled glove box. Approximately 5 mg of CNTs (MWCNTs or SWCNTs) were placed at the end of a silica tube, previously sealed. A notch was made along the tube to define the position of the temperature gradient, 18 cm from the end where the CNTs were located. 200 mg of Bi_2Te_3 and 5 mg of I_2 were ground together using an agate mortar and pestle until a uniform mixture was obtained, which was subsequently pressed into an 8 mm diameter pellet under a load of 2 tons. The pellet was placed inside the silica tube at the position defined by the notch. The silica ampoule was then vacuum sealed at 10^{-4} mbar. The ampoule was placed into a two-zone tubular furnace, setting the temperature at 520 °C ("hot zone") in the area of the Bi_2Te_3–I_2 pellet and at 475 °C ("cold zone") for the CNTs. The target temperature of the hot zone was reached at a heating rate of 200 $°C \cdot h^{-1}$, while the heating rate of the cold zone was adjusted so that both temperature controllers reached their set points simultaneously. To ensure effective material transport from the hot zone to the cold zone, the ratio between

the amount of iodine (m_{I_2}) and the ampoule volume ($V_{ampoule}$) was considered. Previous studies on transport reactions reported that $m_{I_2}/V_{ampoule}$ should not exceed 0.5 mg/mL to promote efficient vapor transport.[2] In this work, the ampoule had a total internal volume of 14 mL, resulting in a $m_{I_2}/V_{ampoule}$ ratio of 0.36 mg/mL. Different temperatures, reaction times, and cooling rates were investigated. The conditions described above correspond to the optimized parameters.

Electron tomography

Electron tomography images were obtained using an FEI Titan[3] microscope, operating at 300 kV. The acquisition parameters for 3D reconstruction were carefully optimized to balance tomographic resolution with the structural integrity of the encapsulated BiTeI. To quantify the influence of the electron dose on the reconstruction quality, two distinct sets of conditions were evaluated. High-resolution volumetric data was obtained for the heterostructure presented in Figure 4, where a pixel size of 48.5 pm and a beam current of 50 pA (1 μs dwell time) resulted in a cumulative electron dose of 1327 $e^-/Å^2$. The tilt-series for this sample spanned a range from –60° to +64° with a 2° increment. The sample shown in Figure S7 was acquired using a lower electron dose of 656 $e^-/Å^2$, achieved by increasing the pixel size to 68.6 pm, with a tilt range from –66° to +72°. In both cases, the 3D volume was reconstructed using the expectation-maximization (EM) algorithm.

Separation of empty and filled SWCNTs by density gradient ultracentrifugation (DGU)

SWCNTs and BiTeI@SWCNT samples were independently dispersed in a solution of 1% sodium deoxycholate (DOC, Acros Organics, 99%) in D_2O (Cortectnet, 99.8 atom%D) under magnetic stirring. The process was carried out at room temperature over two weeks, achieving adequate dispersion. Afterwards, the dispersions were centrifuged at 14,000

rpm for 2 h (with a tabletop centrifuge Sigma 2-16 KCH equipped with a swing-out rotor, 16,215g). The supernatant (a light-grey fraction) containing individually dispersed nanotubes was collected, whereas the black sediment at the bottom corresponded to undispersed bundles. The grey supernatant was carefully extracted and transferred into a density gradient medium composed of a mixture of Nycodenz (iohexol, Axis-Shield) and 1% DOC/D_2O, forming two layers of low (ρ = 1.186 g/mL) and high density (ρ = 1.265 g/mL) that were added on top of each other with a pipette.[28] Then, the centrifuge tubes were tilted and rotated around their axis to obtain a quasi-linear gradient before starting the ultracentrifugation. Ultracentrifugation was carried out at 46,000 rpm (236,400 g) for 20 h (Beckman Coulter Optima max-XP, equipped with MLS-50 swing-out rotor, at 20 °C), after which five distinct fractions were carefully collected with a syringe. Fractions A, D, and E were discarded due to the absence of SWCNTs in fraction A, and the presence of undispersed bundles in fractions D and E.[3,4] Fractions B and C, which contained well-dispersed SWCNTs, were dialyzed against 1% DOC/D_2O to remove the Nycodenz and concentrate the samples using an Amikon stirred ultrafiltration cell equipped with a 25 mm large 100 kDa ultrafiltration disc (Millipore). The same separation procedure was performed for the reference (empty) SWCNT received sample to enable direct comparison with BiTeI@SWCNTs.

Characterization

The morphology and distribution of the inorganic structures grown within the CNTs was determined by high-resolution transmission electron microscopy (HRTEM) and high-angle annular dark field (HAADF) imaging in scanning transmission electron microscopy (STEM) mode. HRTEM images were obtained using a probe-corrected Thermo Fisher Scientific Spectra 300 (S)TEM microscope, operating at 200 kV, and an image-corrected FEI Titan^3 microsope operating at 300 kV. Energy-dispersive X-ray spectroscopy (EDX)

and electron energy loss spectroscopy (EELS) analysis was perfomed using a Thermo Fisher Scientific Spectra 300 (S)TEM microscope. Samples were prepared by sonicating and dispersing a small amount of powder in hexane. The dispersed samples were placed dropwise onto lacey carbon Cu grids.

The filling yield was quantitatively determined by a linear intercept method using the ImageJ software on a representative set of HAADF-STEM images. For each micrograph, the total cumulative length of the observed MWCNTs (L_{total}) and the individual lengths of the encapsulated BiTeI nanowires (L_{fill}) were measured. The filling fraction was then calculated as the ratio of the total encapsulated length to the total nanotube length ($\sum L_{fill}/\sum L_{total}$ x 100).

X-ray Photoelectron Spectroscopy (XPS) analysis was perfomed on a Kratos Axis Supra with Mono Al Kα 120W (8mA/15kV) X-ray source.

Raman experiments were carried out in a DilorXY800 triple Raman spectrometer equipped with a liquid nitrogen-cooled SiCCD. Samples were excited with an Argon ion laser at 514.5 nm.

Near-infrared (NIR) spectra to determine the optical band gap (E_g) was recorded with a Bruker Vertex 70 FTIR spectrophotometer coupled to a Hyperion (Bruker) optical microscope. The optical E_g uncertainties for both pristine carbon nanotubes (MWCNTs and SWCNTs) and the resulting heterostructures (BiTeI@MWCNTs and BiTeI@SWCNTs) were determined by performing multiple measurements at different positions on the KBr pellets to account for sample heterogeneity. For the isolated BiTeI, the E_g was derived from the net absorbance spectra obtained after subtracting the scaled reference signal. The associated error was calculated through the propagation of the standard errors of the intercept and the slope from the linear fit of the Tauc plots.

Preparation and characterization of thermoelectric samples

SWCNTs and BiTeI@SWCNTs samples were independently dispersed at a concentration of 0.1 mg/mL in water containing 1 mg/mL of sodium dodecylbenzenesulfonate (SDBS) as surfactant and using 5 min of tip-sonication. Dispersions were vacuum filtered onto PTFE membranes with 100 nm pore size, and rinsed with copious amounts of water to remove the SDBS. Electrical conductivity and Seebeck coefficient were measured with a custom-built setup using silver paste contacts placed at the corners of 10x10 mm^2 samples cut from the films. [5] Film thickness, typically of the order of 200 nm, was measured using a KLA Tencor Alpha-Step D-500 profilometer by delaminating the offcuts from the PTFE filter and transferring them to glass slides.

Sample preparation for infrared spectroscopy

Samples for infrared (IR) spectroscopy were prepared as transparent pellets under argon in a glovebox to prevent oxidation and moisture absorption. For each measurement, 1 mg of either empty (SWCNTs or MWCNTs) or filled nanotubes (BiTeI@MWCNT and BiTeI@SWCNT) was ground with 99 mg of anhydrous potassium bromide (KBr) using an agate mortar and pestle until a homogeneous powder was obtained. The resulting mixture was transferred to a 1.3 cm diameter die and pressed at 2 tons for 5 minutes.

Computational methods

We performed density-functional theory (DFT) calculations using VASP[6–9] with projector-augmented wave (PAW) potentials[10,11] of monolayer (1L) and bulk BiTeI. We used a plane-wave energy cutoff of 600 eV, the generalized gradient approximation (GGA) in the parameterization of Perdew–Burke–Ernzerhof (PBE)[12] for the exchange-correlation energy, and a Γ-centered $\boldsymbol{k}$-point mesh of 12×12×1 and 12×12×6 for the 1L and bulk, respectively. We included both spin-orbit coupling (SOC) and van der Waals

interactions, via the non-local optB88-vdW functional of Klimeš *et al.*[13] All geometries were optimized until the residual forces on the atoms were less than 0.001 eV/Å. In the case of the 1L, we used a ~45 Å thick vacuum buffer to avoid spurious interactions with the periodic images along the cross-plane direction. With these computational parameters, we obtained a lattice parameter of 4.3834 Å for the 1L and an in-plane (out-of-plane) of 4.4014 Å (6.9746 Å) for the bulk.

Synthesis of BiTeI@CNTs

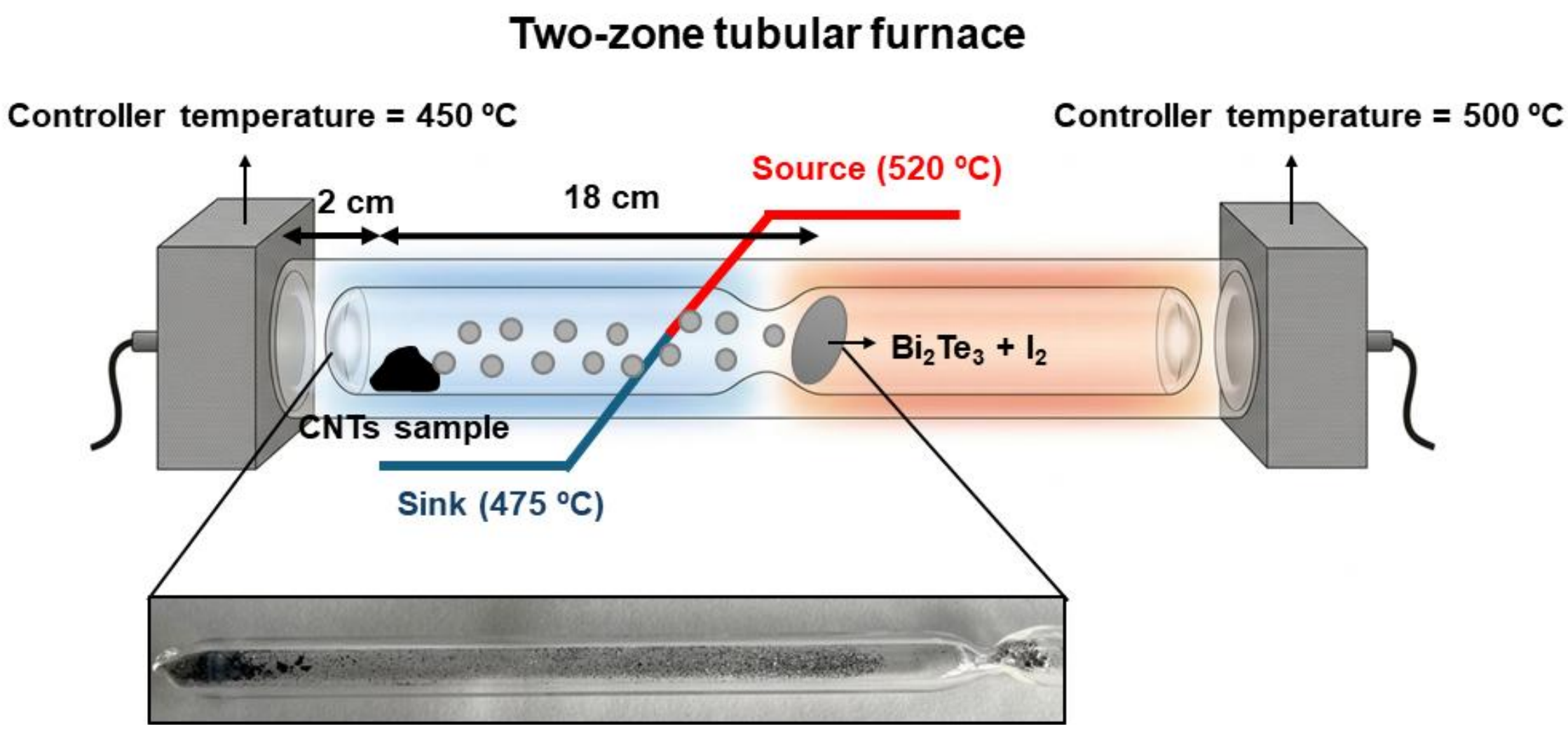

Figure S1. Schematic diagram of the PVT synthesis system and image of the silica tube at the end of the treatment (the picture corresponds to a synthesis using MWCNTs).

BiTeI nanowires were synthesized via sublimation and a subsequent reaction of the precursors (Bi_2Te_3 and I_2), using CNTs as templates (**Figure S1**). When the reaction was performed using the same precursors but in the absence of CNTs, Bi_2Te_3 crystals were obtained instead of the ternary BiTeI compound (**Figure S2**). Although the bulk synthesis of BiTeI via the reaction of Bi_2Te_3 and iodine has been previously reported,[2] this is highly sensitive to both temperature and halogen concentration. In fact, BiTeI is typically synthesized either from BiI_3 and Bi_2Te_3[14,15] or from an equimolar mixture of bismuth, iodine, and tellurium.[16,17]

a

EDX Quantitative Results		
Element	Wt (%)	At (%)
Bi-M	43.76	32.21
Te-L	56.24	67.79

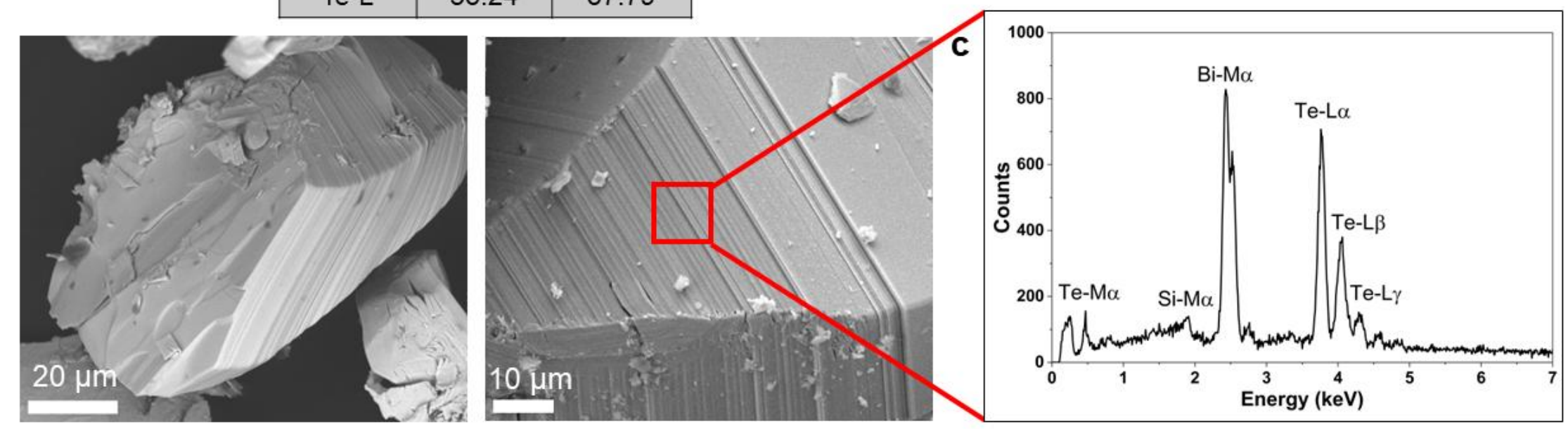

Figure S2. Morphological and chemical characterization of synthesized bulk Bi_2Te_3. (**a**) Table summarizing the weight (wt. %) and atomic (at. %) composition of the sample as determined by EDX. (**b**) SEM images at different magnifications revealing the layered morphology of a bulk Bi_2Te_3 crystal. (**c**) EDX spectrum acquired from the region highlighted by the red box in (b).

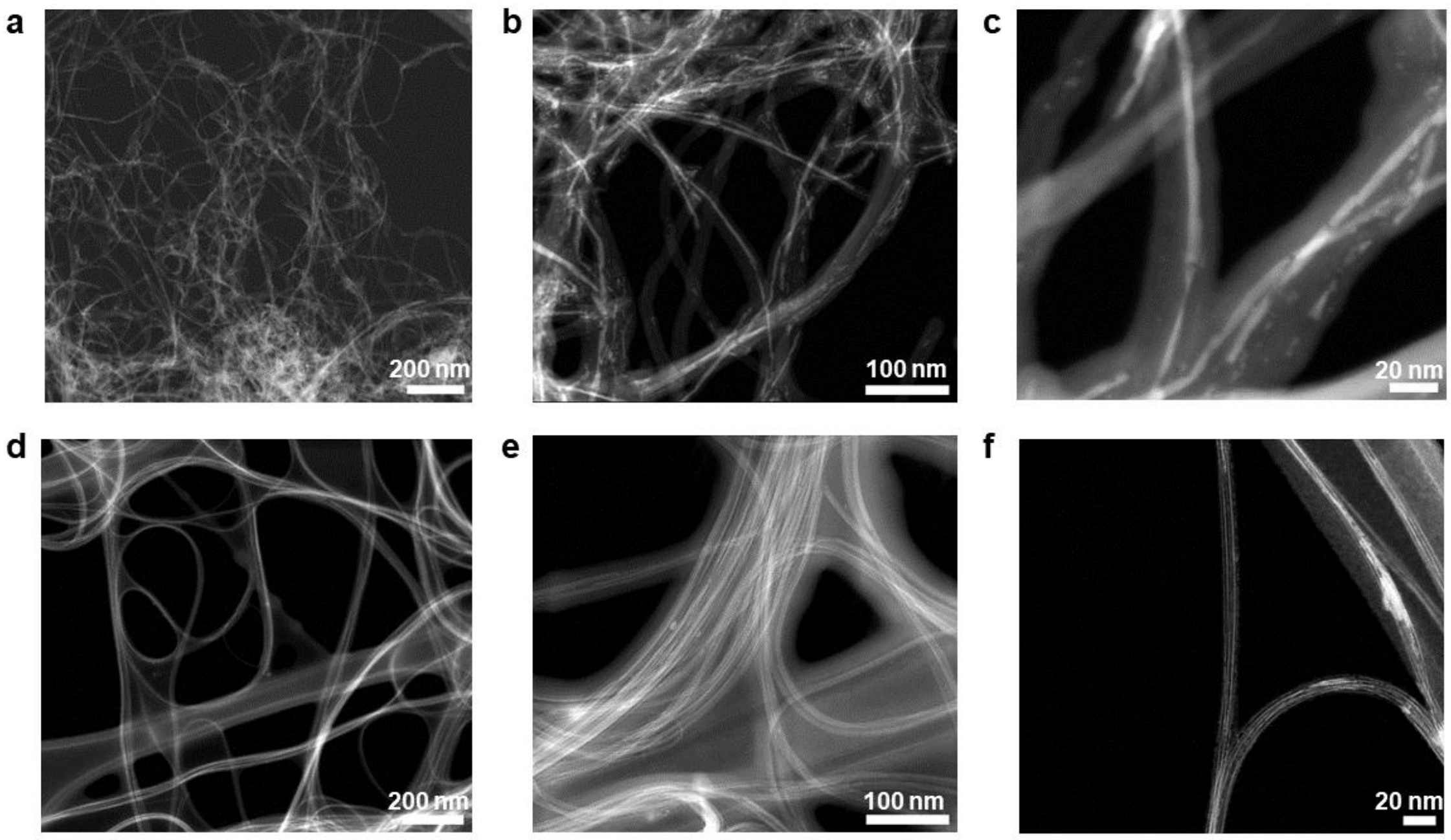

Figure S3. (**a-c**) HAADF-STEM images of BiTeI@MWCNTs. (**d-f**) HAADF-STEM images of BiTeI@SWCNTs. Both types of CNTs reveal a high filling yield.

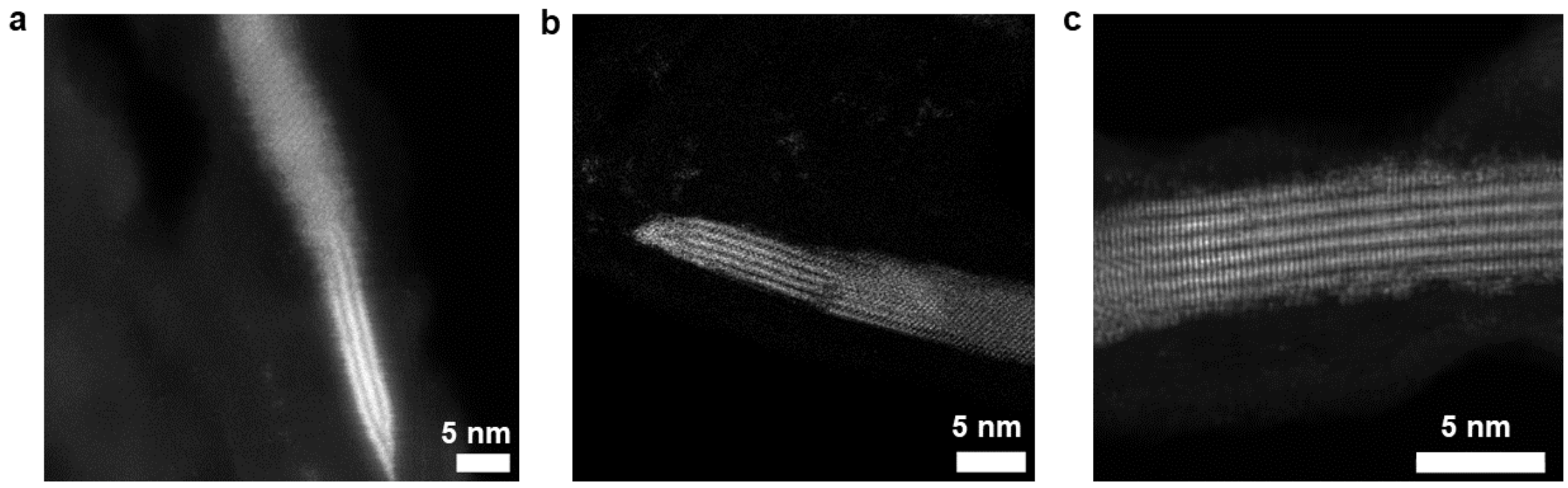

Figure S4. High-resolution HAADF-STEM images of layered BiTeI encapsulated within MWCNTs illustrating the confinement of a varying number of BiTeI atomic layers within the nanotube cores. Nanotube filled with (a) three, (b) five and (c) six discrete BiTeI layers.

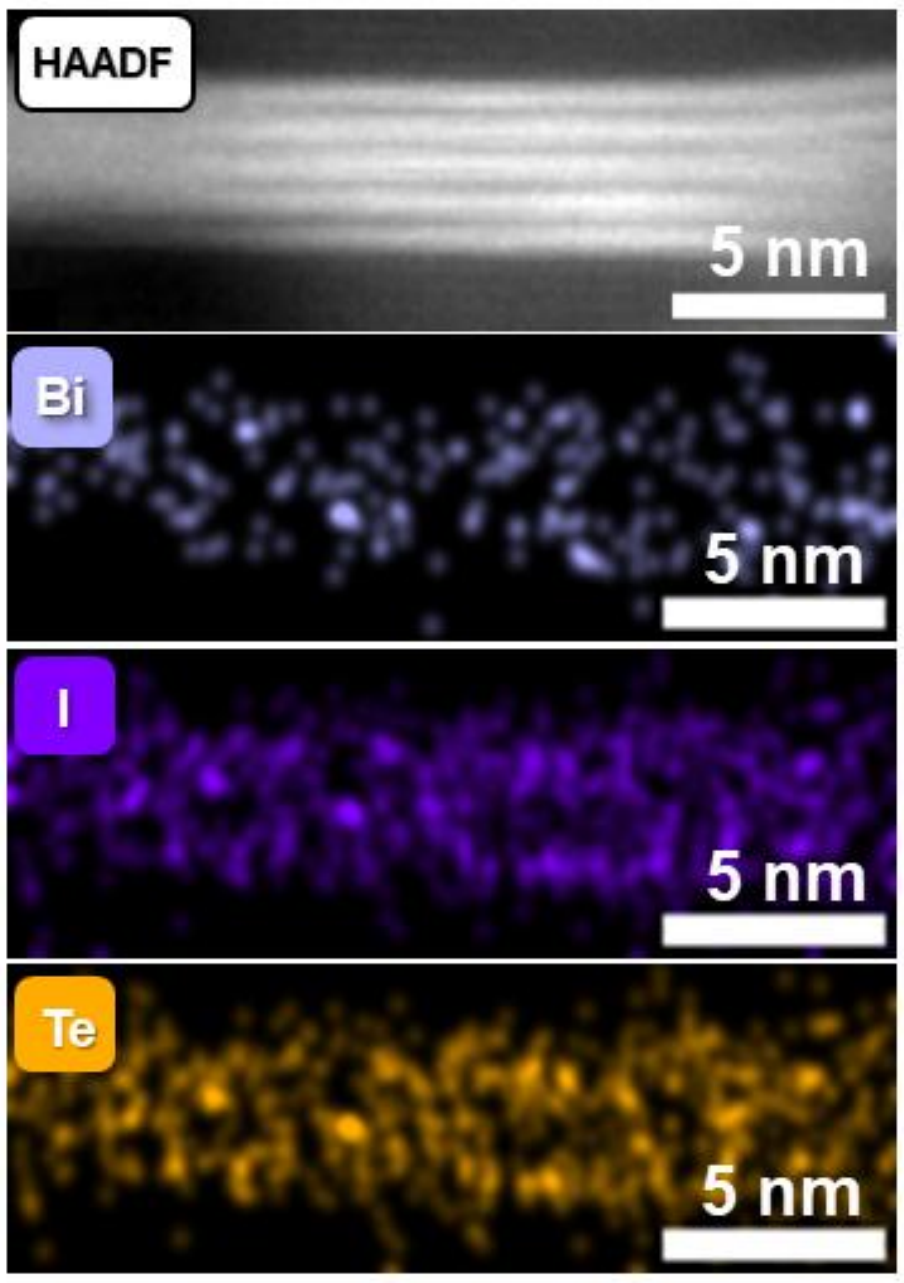

Figure S5. HAADF-STEM image of BiTeI@MWCNT along with the corresponding EDX elemental mapping of Bi (light violet), I (purple) and Te (orange) showing the spatial distribution of the encapsulated material.

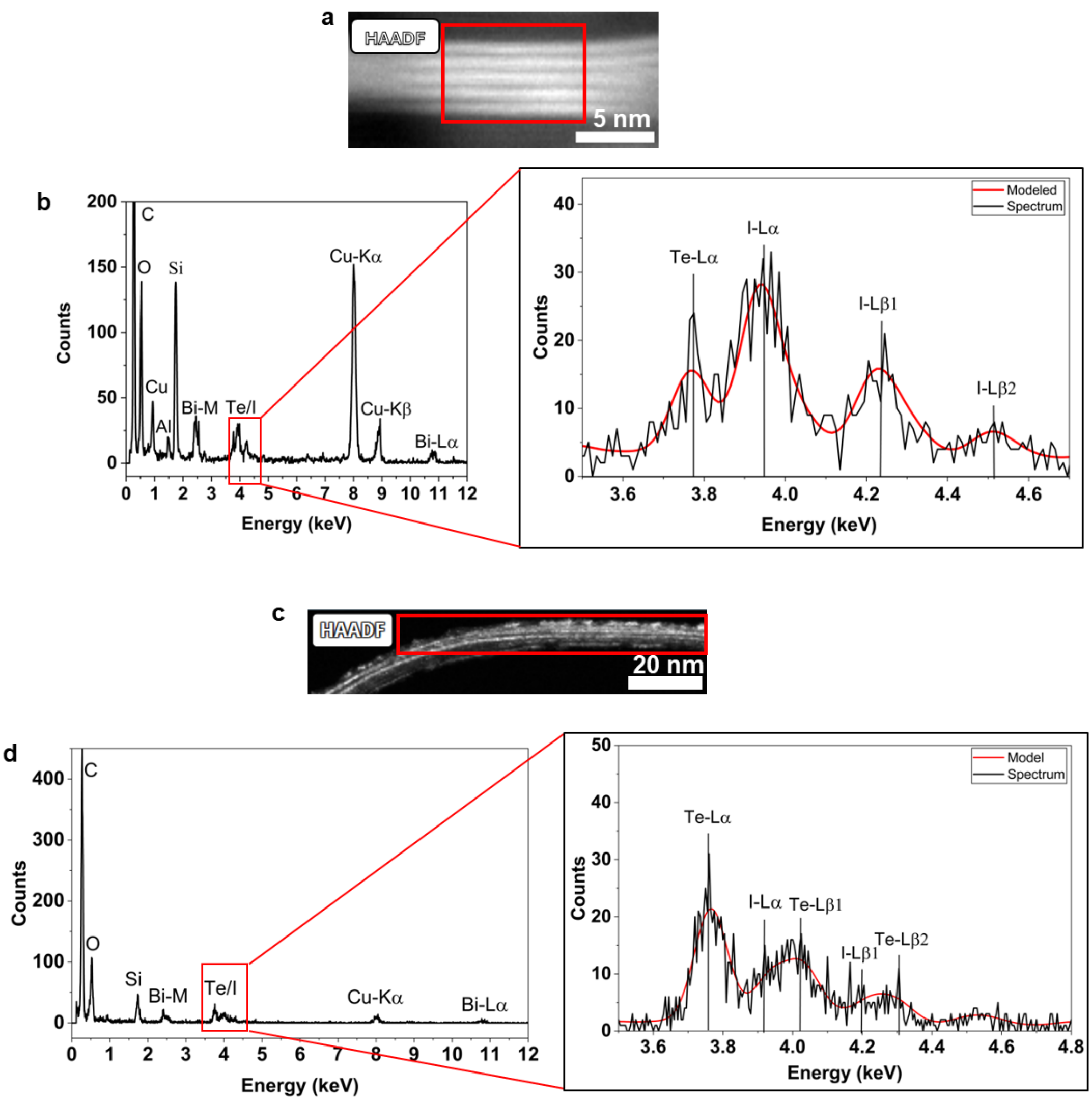

Figure S6. (**a**) HAADF-STEM image of BiTeI@MWCNT and (**b**) EDX spectrum from the highlighted region of BiTeI@MWCNT, confirming the presence of Bi, Te, and I within the MWCNT cavity. (**c**) HAADF-STEM image of BiTeI@SWCNT and (**d**) EDX spectrum from the highlighted region of BiTeI@SWCNT, confirming the presence of Bi, Te, and I within the MWCNT cavity. The C, Si, O, and Cu signals arise from the CNT walls, the silica tube, and the TEM grid, respectively.

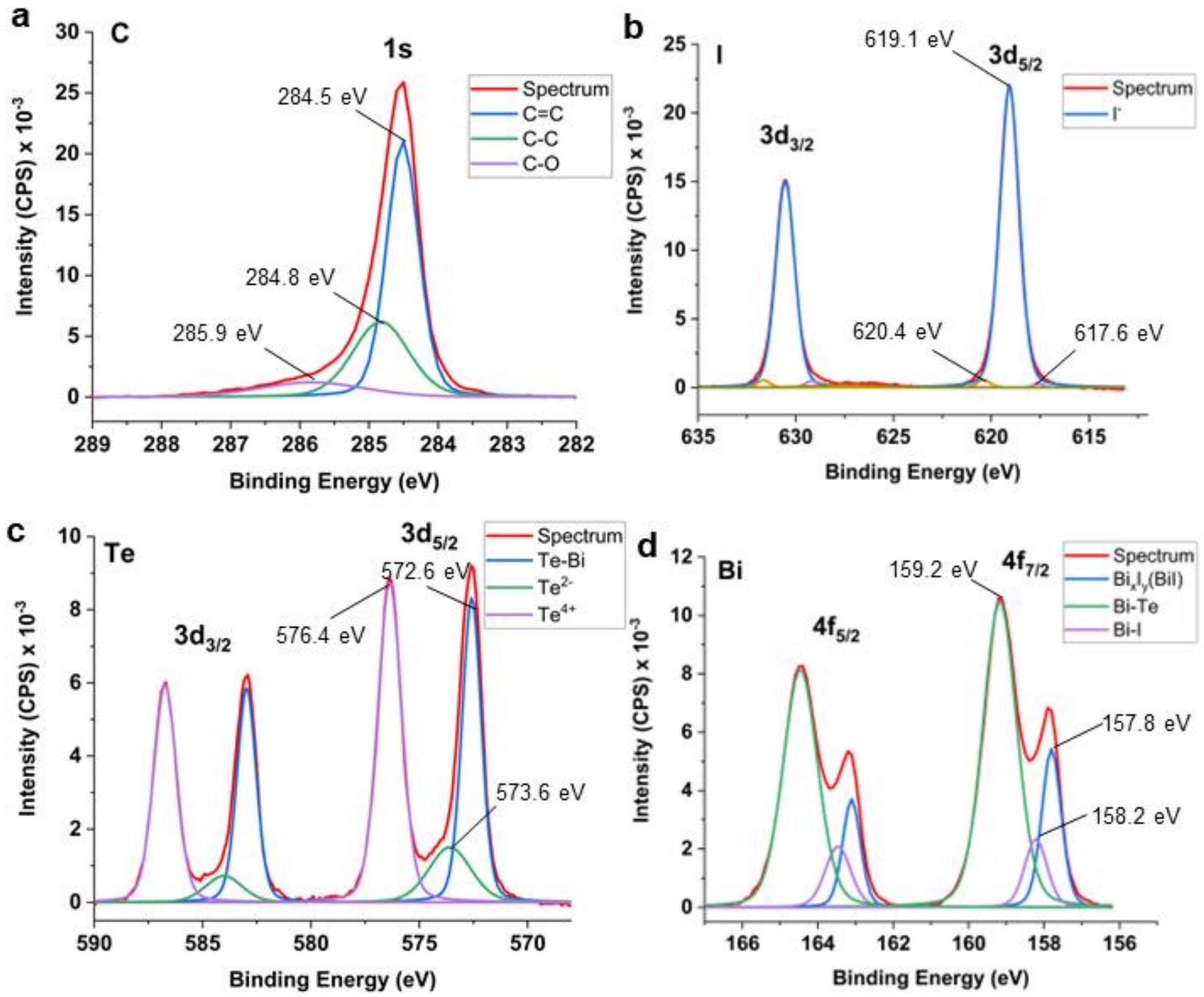

Figure S7. XPS spectra of BiTeI@MWCNT. (**a**) C 1s region, (**b**) I 3d region with peaks at 619.1 eV and 630.6 eV confirming the presence of iodide. (**c**) Te 3d region with Te $3d_{5/2}$(572.6 eV) and Te $3d_{3/2}$ (576.4 eV) peaks along with a minor oxide component. (**d**) Bi 4f region with Bi $4f_{7/2}$ (157.8 eV) and Bi $4f_{5/2}$ (159.2 eV) peaks, consistent with the presence of Bi^{3+} in BiTeI. The sp^2 carbon peak was used as a reference for calibration of the recorded spectra.

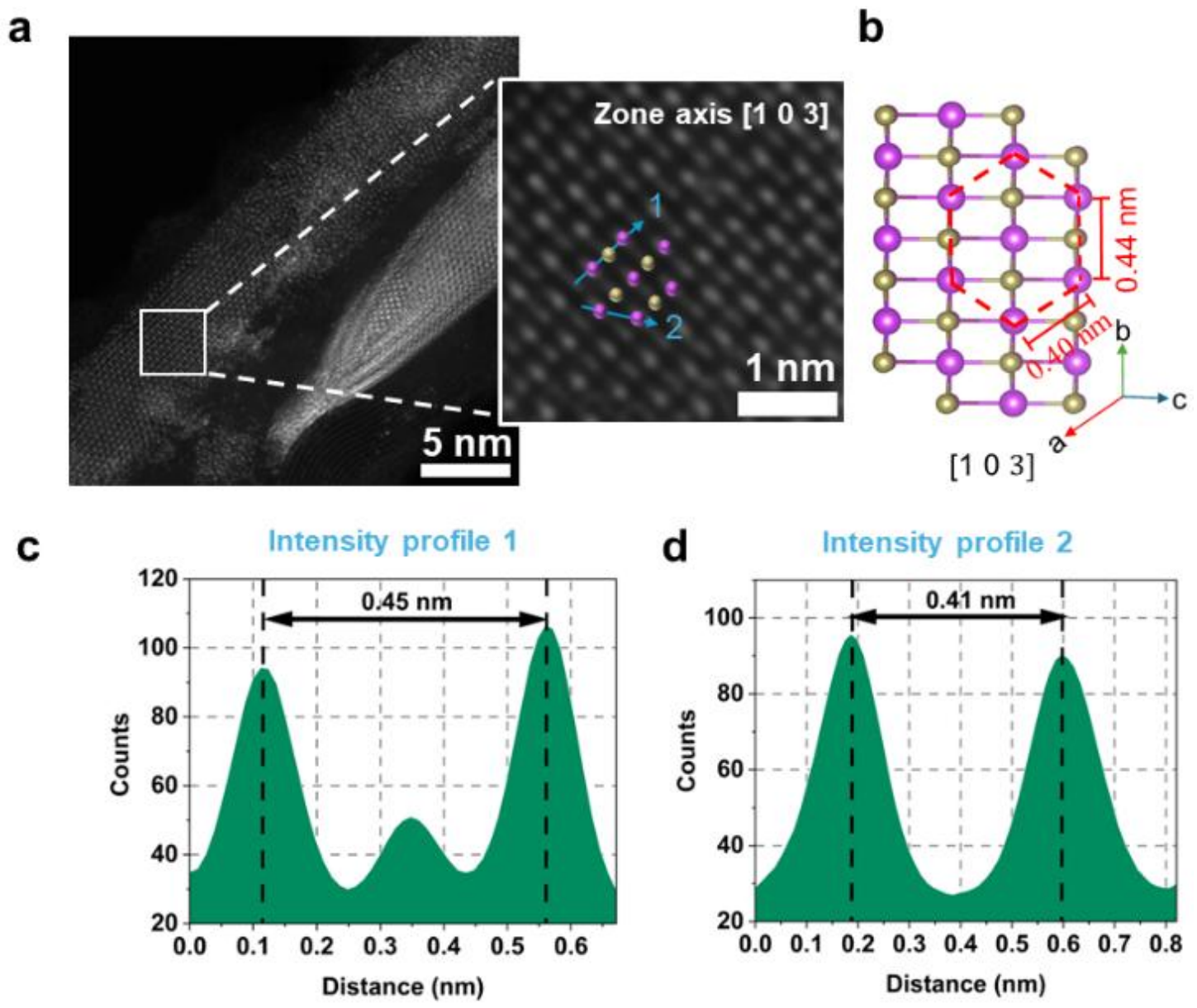

Figure S8. Structural characterization of a BiTeI nanowire encapsulated within a MWCNT. (**a**) HAADF-STEM image of BiTeI@MWCNT. Bi and Te/I positions are represented by violet and ochre spheres, respectively. (**b**) Bulk crystal structure of BiTeI viewed along the [1 0 3] zone axis.[18] Bi, Te and I atoms are represented by violet, ochre and purple spheres, respectively. Note that I atoms are barely visible in this representation because they share the same atomic columns than Te. (**c**, **d**) Intensity profiles along blue arrows 1 and 2 (in image a).

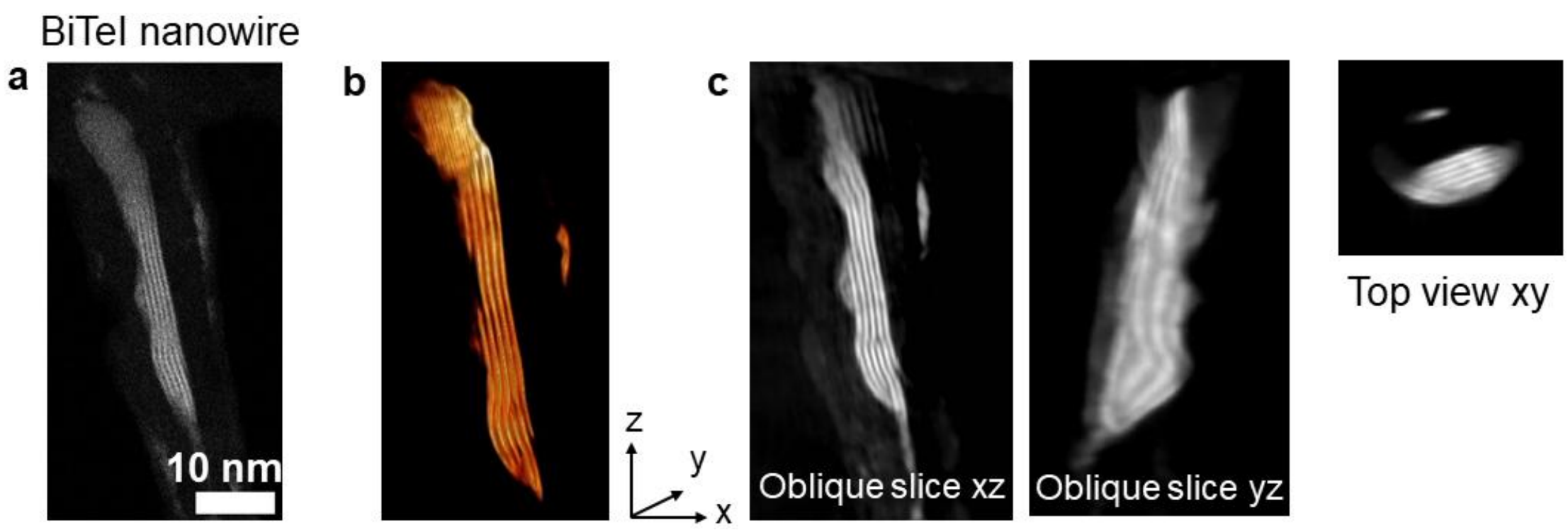

Figure S9. Electron tomography reconstruction of a BiTeI nanowire encapsulated within an MWCNT. (**a**) HAADF-STEM projected images obtained prior to tomographic reconstruction. (**b**) Corresponding 3D reconstructions showing the internal structure and morphology of the encapsulated material. (**c**) Oblique slices of the tomographic volumes along the yz, xz, and xy planes, revealing the spatial distribution and morphology of BiTeI within the MWCNTs cavity.

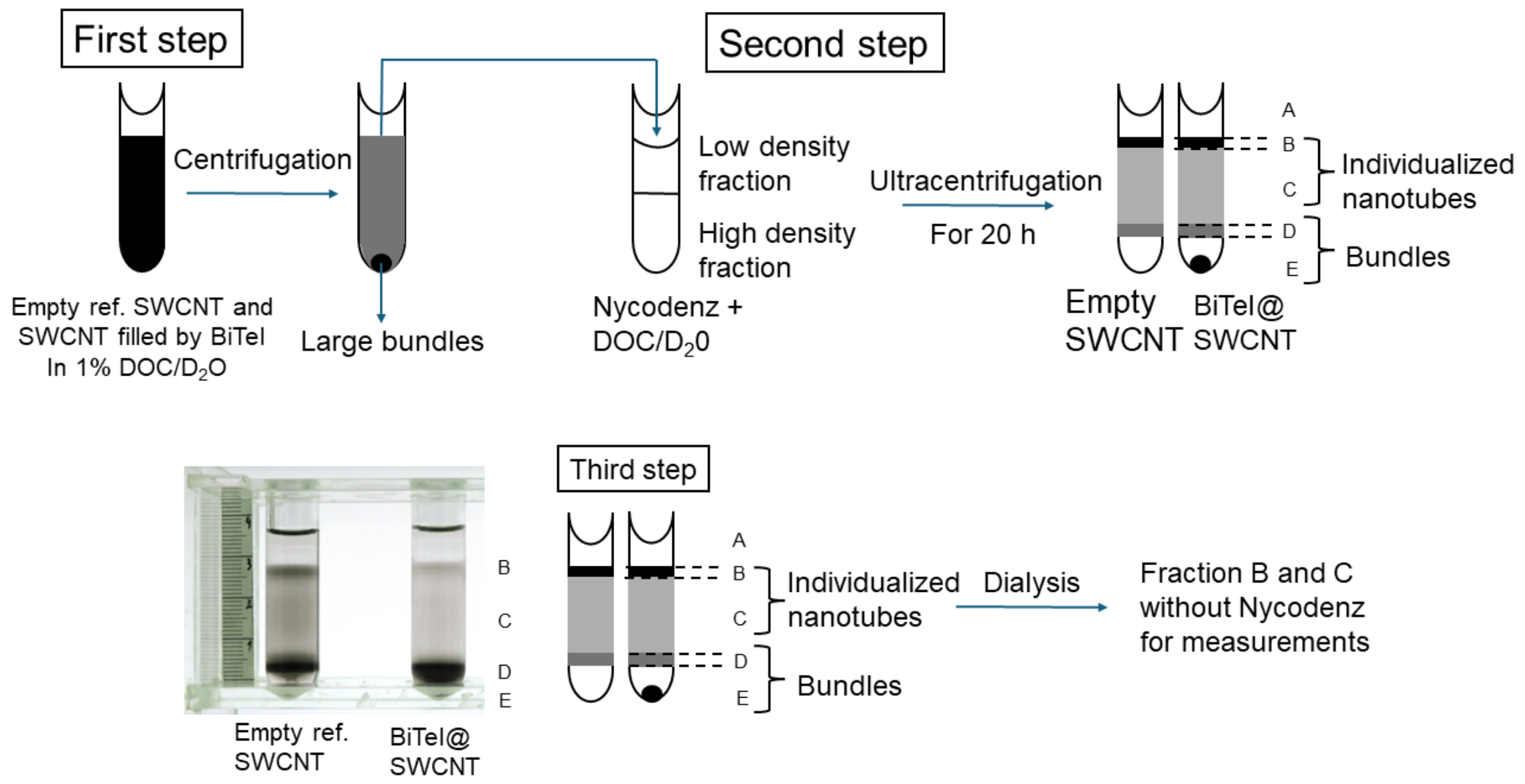

Figure S10. Schematic diagram of the DGU process. The separation of different phases can be observed for both the reference sample (empty SWCNTs) and the sample filled with BiTeI.

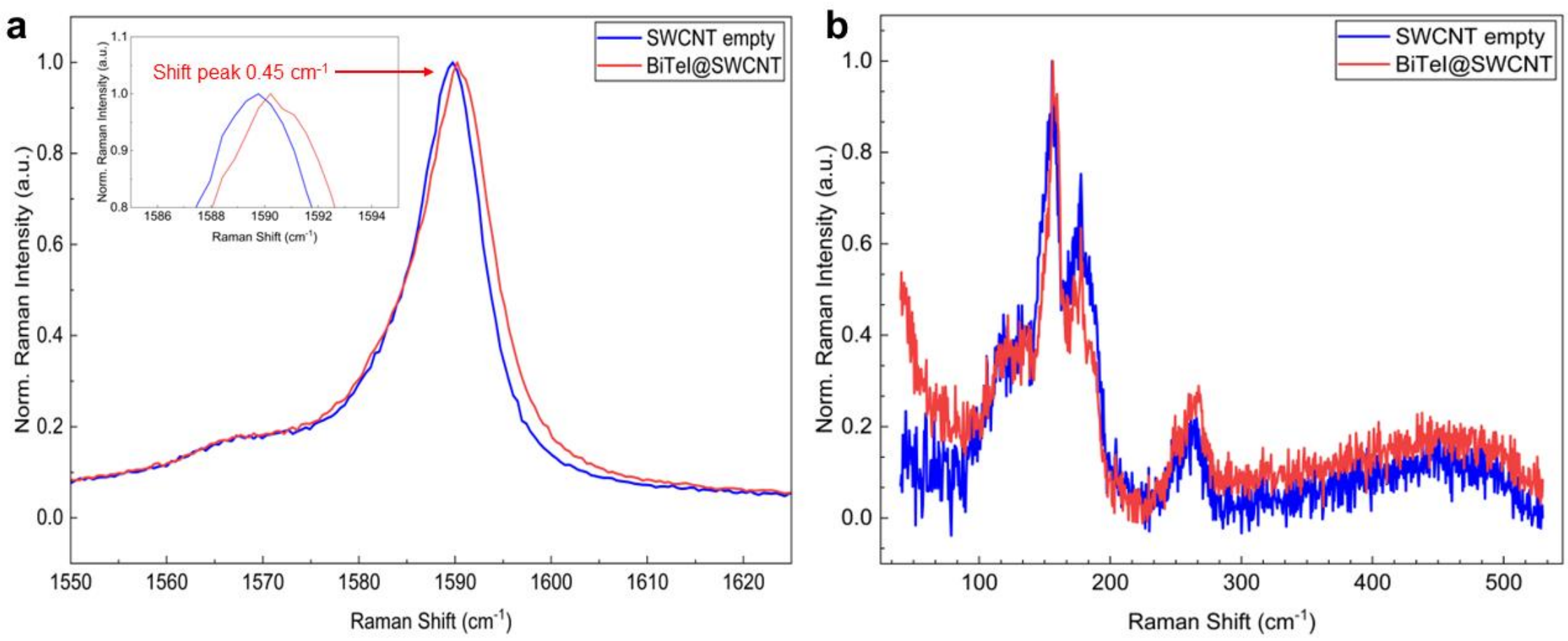

Figure S11. Raman spectra of empty SWCNTs and BiTeI@SWCNTs before DGU process. (**a**) G-band region (514.5 nm excitation) showing a peak upshift of ~0.45 cm^{-1} after filling, indicative of charge transfer or strain effects. (**b**) RBM region (514.5 nm excitation) where no significant new peaks are distinguished.

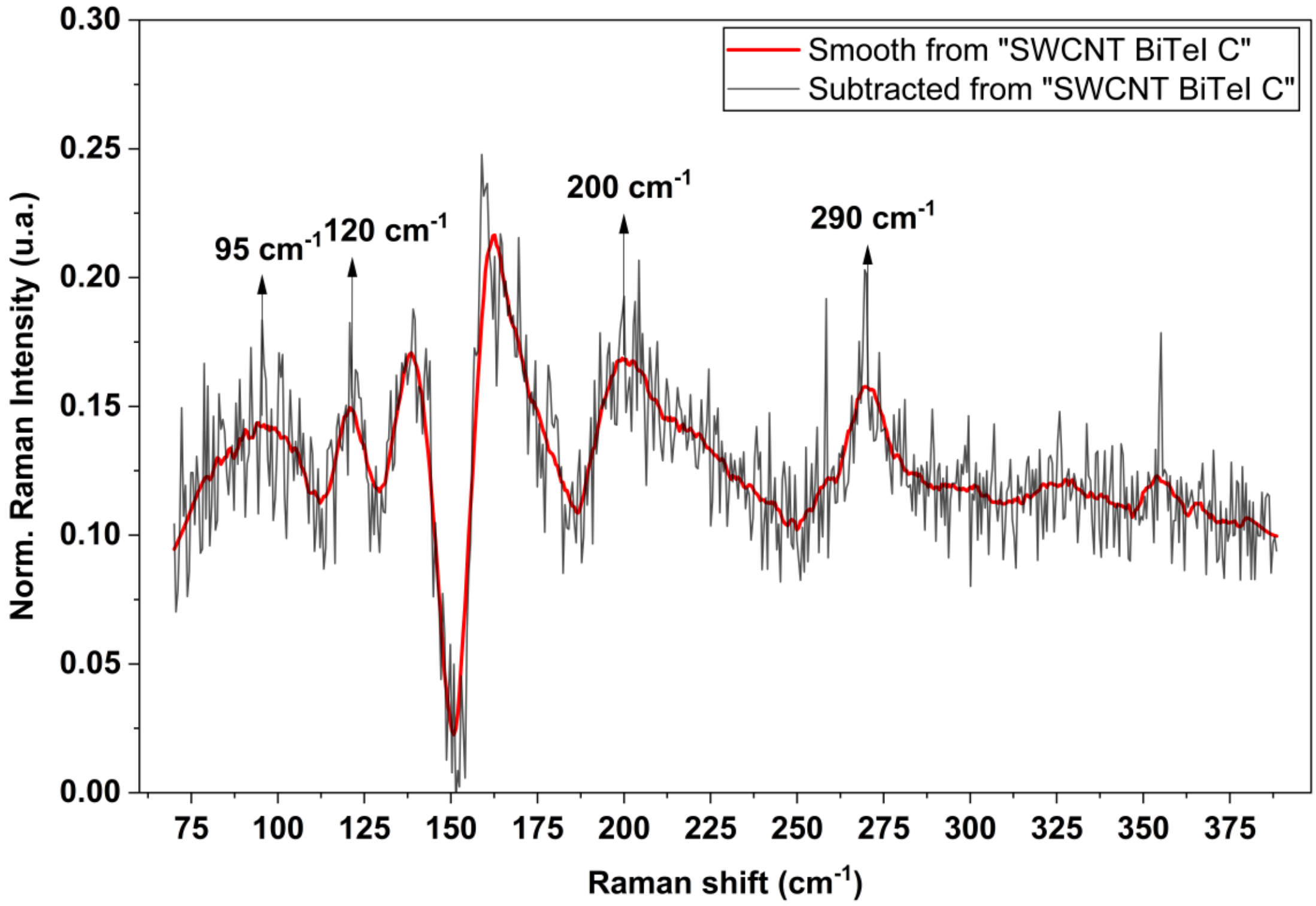

Figure S12. Raman spectrum of the radial breathing mode (RBM) region obtained by subtracting the pristine SWCNT reference from the BiTeI@SWCNT (fraction C) sample.

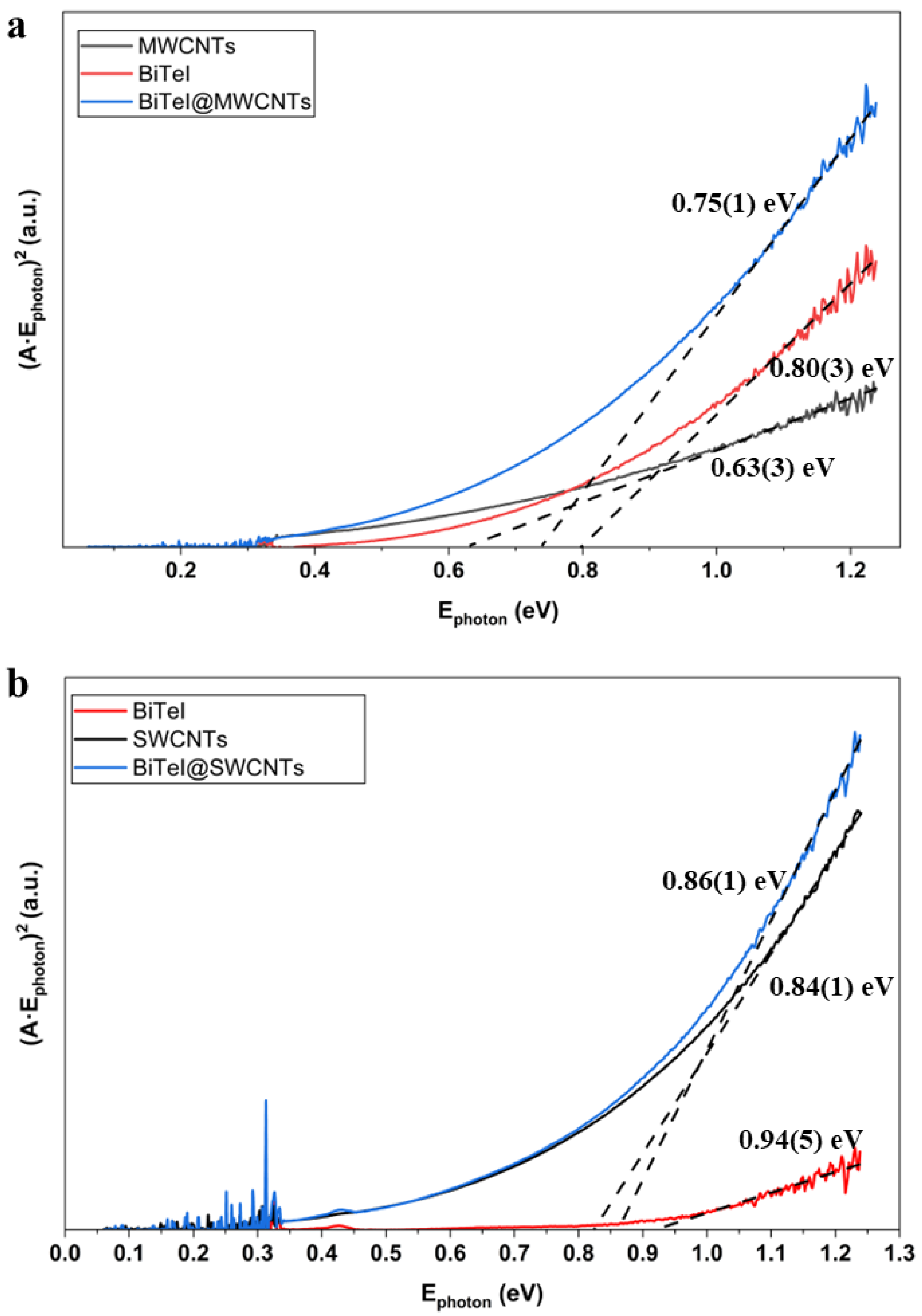

Figure S13. Tauc plots of $(A\cdot Ephoton)^2$ for (**a**) MWCNTs and (**b**) SWCNTs samples. Here, E_{photon} and A denote the photon energy and absorbance in optical absorption measurements.

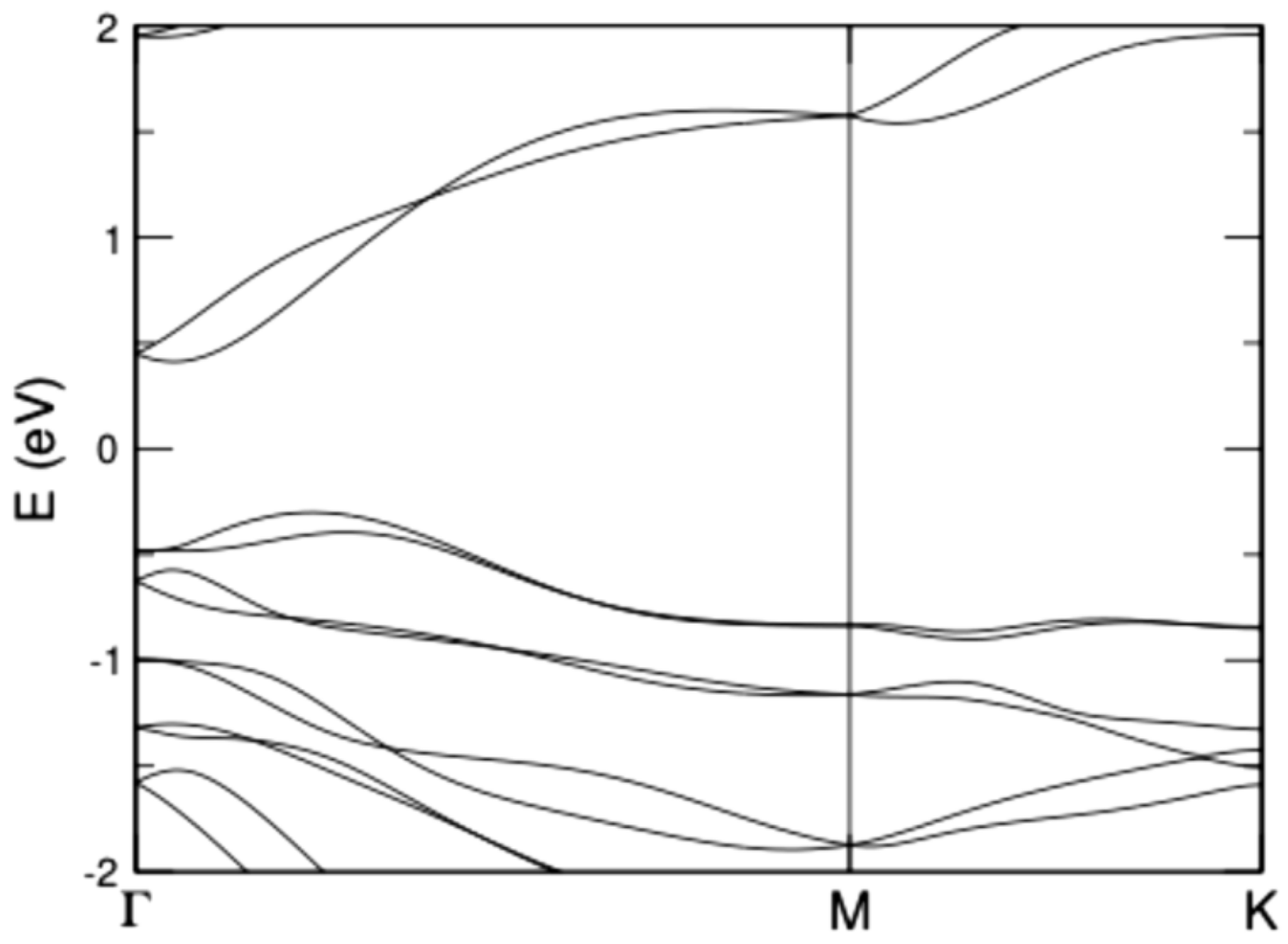

Figure S14. Full electronic band structure of single-layer BiTeI where Γ= (0, 0), M= (*a**/2, 0) and K= (*a**/3, *a**/3).

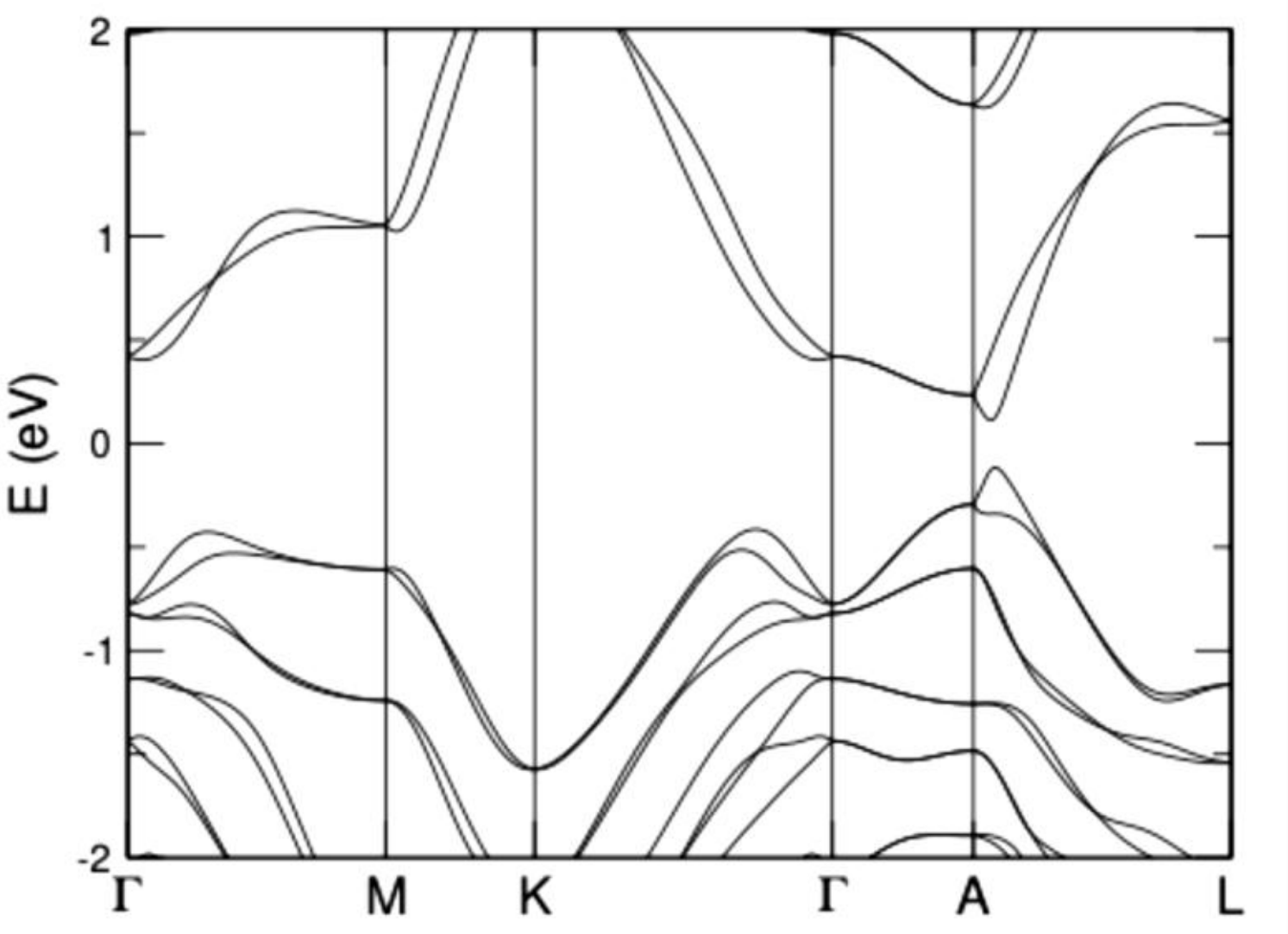

Figure S15. Full electronic band structure of bulk BiTeI where Γ= (0, 0, 0), M= (*a**/2, 0, 0), K= (*a**/3, *a**/3, 0), A= (0, 0, *c**/2), L= (a*/2, 0, *c**/2).

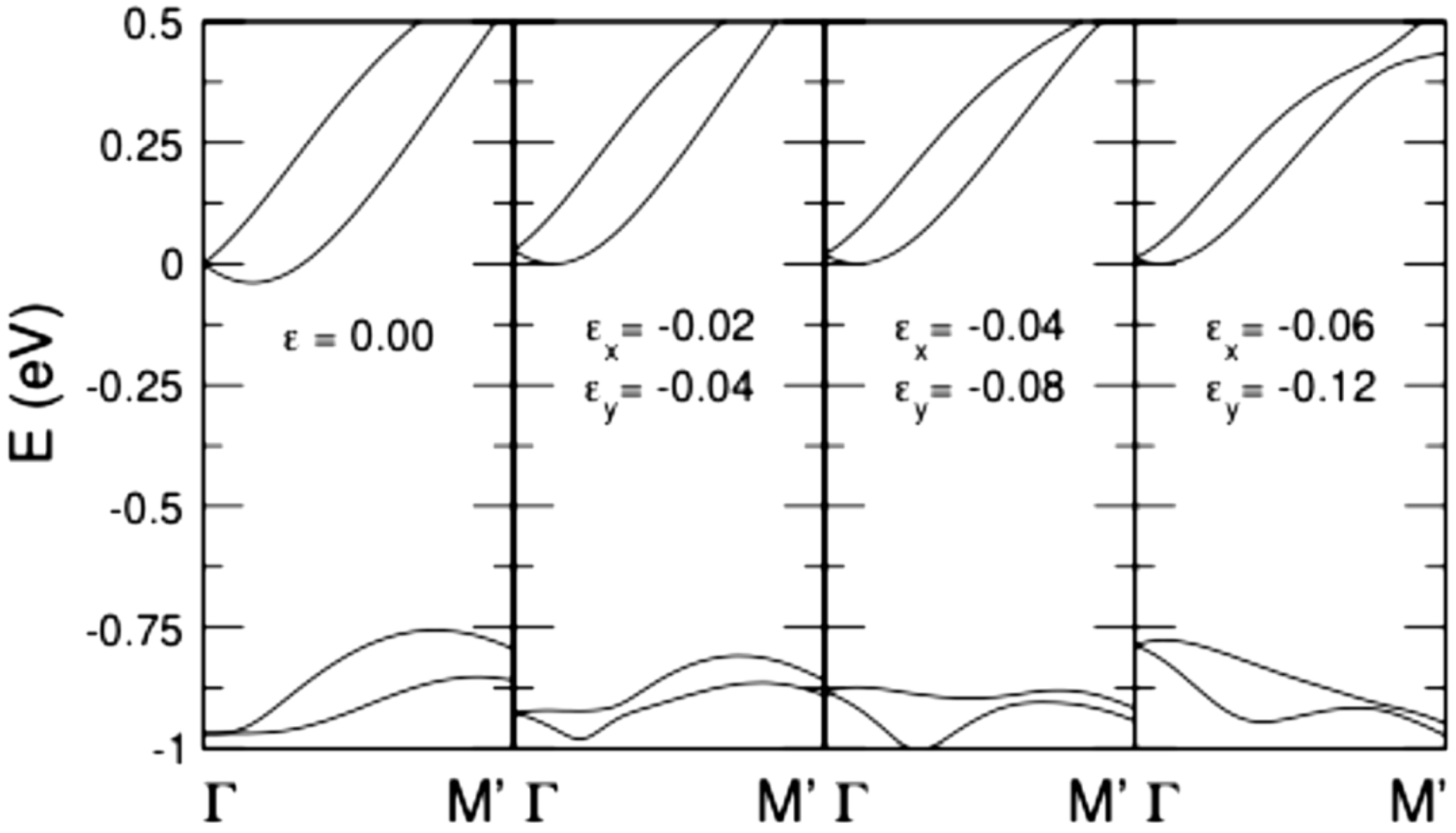

Figure S16. Evolution of the bottom/top part of the conduction/valence band of a BiTeI single-layer as a function of non-uniform strain along the Γ to M direction. Γ= (0,0), M’= (*a**/6,0).

REFERENCES

(1) Cabana, L.; Ke, X.; Kepić, D.; Oro-Solé, J.; Tobías-Rossell, E.; Van Tendeloo, G.; Tobias, G. The Role of Steam Treatment on the Structure, Purity and Length Distribution of Multi-Walled Carbon Nanotubes. *Carbon N. Y.* **2015**, *93*, 1059–1067. https://doi.org/10.1016/j.carbon.2015.06.027.

(2) Schöneich, M.; Schmidt, M. P.; Schmidt, P. Chemical Vapour Transport of Bismuth and Antimony Chalcogenides M2Q3 (M = Sb, Bi, Q = Se, Te). *Z. Anorg. Allg. Chem.* **2010**, *636* (9–10), 1810–1816. https://doi.org/10.1002/zaac.201000149.

(3) Cui, J.; Yang, D.; Zeng, X.; Zhou, N.; Liu, H. Recent Progress on the Structure Separation of Single-Wall Carbon Nanotubes. *Nanotechnology* **2017**, *28* (45), 452001. https://doi.org/10.1088/1361-6528/aa8ac9.

(4) Erkens, M.; Levshov, D.; Wenseleers, W.; Li, H.; Flavel, B. S.; Fagan, J. A.; Popov, V. N.; Avramenko, M.; Forel, S.; Flahaut, E.; Cambré, S. Efficient Inner-to-Outer Wall Energy Transfer in Highly Pure Double-Wall Carbon Nanotubes Revealed by Detailed Spectroscopy. *ACS Nano* **2022**, *16* (10), 16038–16053. https://doi.org/10.1021/acsnano.2c03883.

(5) Dörling, B.; Zapata-Arteaga, O.; Campoy-Quiles, M. A Setup to Measure the Seebeck Coefficient and Electrical Conductivity of Anisotropic Thin-Films on a Single Sample. *Review of Scientific Instruments* **2020**, *91* (10), 105111. https://doi.org/10.1063/5.0021715.

(6) Kresse, G.; Hafner, J. Ab Initio Molecular Dynamics for Liquid Metals. *Phys. Rev. B* **1993**, No. 47. https://doi.org/10.1103/PhysRevB.47.558.

(7) Kresse, G.; Hafner, J. Ab Initio Molecular-Dynamics Simulation of the Liquid-Metal--Amorphous-Semiconductor Transition in Germanium. *Phys. Rev. B* **1994**, *49* (20), 14251–14269. https://doi.org/10.1103/PhysRevB.49.14251.

(8) Kresse, G.; Furthmüller, J. Efficient Iterative Schemes for Ab Initio Total-Energy Calculations Using a Plane-Wave Basis Set. *Phys. Rev. B* **1996**, *54* (16), 11169–11186. https://doi.org/10.1103/PhysRevB.54.11169.

(9) Kresse, G.; Furthmüller, J. Efficiency of Ab-Initio Total Energy Calculations for Metals and Semiconductors Using a Plane-Wave Basis Set. *Comput. Mater. Sci.* **1996**, *6* (1), 15–50. https://doi.org/10.1016/0927-0256(96)00008-0.

(10) Blöchl, P. E. Projector Augmented-Wave Method. *Phys. Rev. B* **1994**, *50* (24), 17953–17979. https://doi.org/10.1103/PhysRevB.50.17953.

(11) Kresse, G.; Joubert, D. From Ultrasoft Pseudopotentials to the Projector Augmented-Wave Method. *Phys. Rev. B* **1999**, *59* (3), 1758–1775. https://doi.org/10.1103/PhysRevB.59.1758.

(12) Perdew, J. P.; Burke, K.; Ernzerhof, M. Generalized Gradient Approximation Made Simple. *Phys. Rev. Lett.* **1996**, *77* (18), 3865–3868. https://doi.org/10.1103/PhysRevLett.77.3865.

(13) Klimeš, J.; Bowler, D. R.; Michaelides, A. Chemical Accuracy for the van Der Waals Density Functional. *Journal of Physics: Condensed Matter* **2010**, *22* (2), 022201. https://doi.org/10.1088/0953-8984/22/2/022201.

(14) Aliev, Z. S.; Ahmadov, E. C.; Babanly, D. M.; Amiraslanov, I. R.; Babanly, M. B. The Bi2Se3–Bi2Te3–BiI3 System: Synthesis and Characterization of the BiTe1-XSexI Solid Solutions. *Calphad* **2019**, *66*, 101650. https://doi.org/10.1016/j.calphad.2019.101650.

(15) Sankar, R.; Panneer Muthuselvam, I.; Butler, C. J.; Liou, S.-C.; Chen, B. H.; Chu, M.-W.; Lee, W. L.; Lin, M.-T.; Jayavel, R.; Chou, F. C. Room Temperature Agglomeration for the Growth of BiTeI Single Crystals with a Giant Rashba Effect. *CrystEngComm* **2014**, *16* (37), 8678–8683. https://doi.org/10.1039/c4ce01006j.

(16) Shevelkov, A. V; Dikarev, E. V; Shpanchenko, R. V; Popovkin, B. A. Crystal Structures of Bismuth Tellurohalides BiTeX (X = Cl, Br, I) from X-Ray Powder Diffraction Data. *J. Solid State Chem.* **1995**, *114* (2), 379–384. https://doi.org/10.1006/jssc.1995.1058.

(17) Antonatos, N.; Kovalska, E.; Mazánek, V.; Veselý, M.; Sedmidubský, D.; Wu, B.; Sofer, Z. Electrochemical Exfoliation of Janus-like BiTeI Nanosheets for Electrocatalytic Nitrogen Reduction. *ACS Appl. Nano Mater.* **2021**, *4* (1), 590–599. https://doi.org/10.1021/acsanm.0c02860.

(18) Kulbachinskii, V. A.; Kytin, V. G.; Kudryashov, A. A.; Kuznetsov, A. N.; Shevelkov, A. V. On the Electronic Structure and Thermoelectric Properties of BiTeBr and BiTeI Single Crystals and of BiTeI with the Addition of BiI3 and CuI. *J. Solid State Chem.* **2012**, *193*, 154–160. https://doi.org/10.1016/j.jssc.2012.05.037.
